# Strategies to inhibit FGFR4 V550L-driven rhabdomyosarcoma

**DOI:** 10.1038/s41416-022-01973-6

**Published:** 2022-09-12

**Authors:** Elisa Fiorito, Patrycja Szybowska, Ellen M. Haugsten, Michal Kostas, Geir F. Øy, Antoni Wiedlocha, Sachin Singh, Sigve Nakken, Gunhild M. Mælandsmo, Jonathan A. Fletcher, Leonardo A. Meza-Zepeda, Jørgen Wesche

**Affiliations:** 1 Department of Tumor Biology, Institute for Cancer Research, The Norwegian Radium Hospital, Oslo University Hospital, Montebello, 0379 Oslo, Norway; 2 Centre for Cancer Cell Reprogramming, Institute of Clinical Medicine, Faculty of Medicine, University of Oslo, Montebello, 0379 Oslo, Norway; 3 Department of Molecular Cell Biology, Institute for Cancer Research, The Norwegian Radium Hospital, Oslo University Hospital, Montebello, 0379 Oslo, Norway; 4 Institute of Medical Biology, Faculty of Health Sciences, The Arctic University of Norway – University of Tromsø, 9037 Tromsø, Norway; 5 Department of Pathology, Brigham and Women’s Hospital, Boston, MA USA; 6 Genomics Core Facility, Department of Core Facilities, Institute for Cancer Research, The Norwegian Radium Hospital, Oslo University Hospital, Montebello, 0379 Oslo, Norway; 7 Department of Molecular Medicine, Institute of Basic Medical Sciences, University of Oslo, 0372 Oslo, Norway

**Keywords:** Growth factor signalling, Paediatric cancer, Oncogenes, Sarcoma

## Abstract

**Background:**

Rhabdomyosarcoma (RMS) is a paediatric cancer driven either by fusion proteins (e.g., PAX3-FOXO1) or by mutations in key signalling molecules (e.g., RAS or FGFR4). Despite the latter providing opportunities for precision medicine approaches in RMS, there are currently no such treatments implemented in the clinic.

**Methods:**

We evaluated biologic properties and targeting strategies for the FGFR4 V550L activating mutation in RMS559 cells, which have a high allelic fraction of this mutation and are oncogenically dependent on FGFR4 signalling. Signalling and trafficking of FGFR4 V550L were characterised by confocal microscopy and proteomics. Drug effects were determined by live-cell imaging, MTS assay, and in a mouse model.

**Results:**

Among recently developed FGFR4-specific inhibitors, FGF401 inhibited FGFR4 V550L-dependent signalling and cell proliferation at low nanomolar concentrations. Two other FGFR4 inhibitors, BLU9931 and H3B6527, lacked potent activity against FGFR4 V550L. Alternate targeting strategies were identified by RMS559 phosphoproteomic analyses, demonstrating that RAS/MAPK and PI3K/AKT are essential druggable pathways downstream of FGFR4 V550L. Furthermore, we found that FGFR4 V550L is HSP90-dependent, and HSP90 inhibitors efficiently impeded RMS559 proliferation. In a RMS559 mouse xenograft model, the pan-FGFR inhibitor, LY2874455, did not efficiently inhibit growth, whereas FGF401 potently abrogated growth.

**Conclusions:**

Our results pave the way for precision medicine approaches against FGFR4 V550L-driven RMS.

## Introduction

Rhabdomyosarcoma (RMS) is the most common malignant soft tissue sarcoma in childhood and adolescence [1–3]. Since RMS cells histologically resemble developing striated skeletal muscle, the main hypothesis suggests that this malignancy features an arrest of myogenesis [1, 4, 5]. As for many paediatric tumours, RMS treatment relies on a combination of surgery, chemotherapy, and radiation therapy [2]. However, despite improvements in the clinical management of the disease, therapy failure still occurs due to drug resistance. The lack of biomarkers and targets has encouraged researchers and clinicians to investigate the molecular biology of RMS [6, 7].

In the last decade, next-generation sequencing and microarray studies on RMS tumours identified specific alterations in genes crucial for normal skeletal muscle development [6]. Upregulation of classical development proteins like Notch and Wnt family members promotes disordered myogenesis and predisposes to RMS [2, 6]. In addition, most RMS harbour genetic alterations inducing aberrant tyrosine kinase pathway activation, including both downstream molecules (e.g., RAS mutations) and upstream receptors [7]. Among these proteins, several studies reported dysregulated expression of the fibroblast growth factor receptor 4 (FGFR4) [7, 8].

FGFR4 is a member of the FGFR family of receptor tyrosine kinases (RTKs) [9]. Upon binding various fibroblast growth factors (FGFs), which serve as ligands, FGFRs dimerise and activate signalling pathways driving proliferation, survival, adhesion, migration and differentiation in different cell types. Developmental studies indicate that FGFR4 is essential for embryonic skeletal muscle myogenesis [10, 11]. During postnatal life, FGFR4 expression is induced during muscle regeneration [12], whereas FGFR4 is typically expressed at low levels in other physiological contexts. Like other FGFR family members, FGFR4 has oncogenic roles in cancer [9]. In RMS, FGFR4 overexpression activates signalling cascades which are associated with aberrations in differentiation and cell survival [13]. In addition, 7.5% of RMS primary tumours bear somatic mutations in the FGFR4 tyrosine kinase domain [8]. Recurrent aberrations include the FGFR4 N535K and V550L/M/E mutations that have been found to autoactivate the receptor [14]. Recent studies indicated that mutant FGFR4 promotes proliferation and metastasis [8]. Subsequently, RMS cells can become dependent on mutant FGFR4 expression for their growth and migration. This suggests possibilities for using mutant FGFR4 as a target in RMS [15].

In the last few years, numerous FGFR inhibitors have been developed for the treatment of cancer [16]. Unfortunately, targeting FGFR4 has encountered challenges [17]. First, many of the inhibitors had intolerable side effects in early phase trials and therefore never reached phase III clinical trials. Second, most of these inhibitors have a lower structural affinity for FGFR4 compared to the other FGFR family members, and high concentrations are needed to be effective. Third, FGFR4 mutations in the tyrosine kinase domain influence the interaction and efficacy of some of the available inhibitors and make the cells resistant. Recently, FGFR4-specific inhibitors have been developed (e.g., FGF401, BLU9931) [18–20]. These interact with a cysteine residue in the FGFR4 active site that is not present in other FGFRs. Thus, they are very specific for FGFR4 and constitute promising drugs against cancers driven by alterations in FGFR4.

To efficiently investigate response and resistance mechanisms, well-characterised preclinical models are required. Here we characterise an RMS cell line, RMS559, which we show to be oncogenically dependent on mutant FGFR4 signalling. We used this cell line to investigate potential targeted therapy approaches directed against FGFR4. This work is a basis for future clinical testing of FGFR4 targeting in RMS patients.

## Materials and methods

### Antibodies and compounds

The following antibodies were used: rabbit anti-FGFR4 (#8562), mouse anti-phospho-FGF Receptor (pFGFR, Tyr653/654) (#3476), mouse anti-phospho-ERK 1/2 (pERK 1/2, Thr202/Tyr204) (#9106), rabbit anti-p44/42 MAPK (ERK 1/2) (#9102), rabbit anti-phospho-PLCγ1 (pPLCγ, Tyr783) (#14008), mouse anti-phospho-AKT (pAKT, Ser473) (#9271), rabbit anti-AKT (#9272) from Cell Signaling Technology (Leiden, The Netherlands); mouse anti-γ-tubulin (T6557) and rabbit IgG (12-370) from Sigma-Aldrich (St. Louis, MO, USA); mouse anti-PLCγ (#sc-7290) from Santa Cruz Biotechnology (Dallas, TX, USA); mouse anti-EEA1 (610456) and mouse anti-HSP90 (610419) from BD transduction laboratories (Franklin Lakes, NJ, USA), mouse anti-LAMP1 (H4A3) from Developmental studies Hybridoma Bank (Iowa City, IA, USA), mouse anti-PDI (ADI-SPA-891) from Enzo Life Sciences (Farmingdale, NY, USA); sheep anti-TGN46 (AHP500G) from Bio-Rad (Hercules, CA, USA). HRP-Streptavidin (016–030-084) and all secondary antibodies were from Jackson Immuno-Research Laboratories (Cambridgeshire, UK).

EDTA-free protease inhibitor and phosphatase inhibitor tablets were from Roche Diagnostics (Basel, Switzerland). DyLight 550 NHS Ester, Hoechst 33342 and ProLong Diamond Antifade Mountant (P36961) and Halt Protease and Phosphatase Inhibitor Cocktail (100×) were purchased from Thermo Fisher Scientific (Waltham, MA, USA). Protein G Sepharose 4 Fast Flow beads were purchased from GE Healthcare Life Sciences (Chicago, IL, USA). Biotin, heparin, and formaldehyde (HT5014) were from Sigma-Aldrich. Inhibitors: LY2874455, LY294002, PI-103, PD0325901, U0126-EtOH, RO5126766, TAK733, TAK632, Roblitinib (FGF401), H3B6527 and BLU9931 were purchased from SelleckChem (Munich, Germany). FGF1 was prepared as previously described [21]. FGF1 labelling with DyLight 550 NHS Ester was performed according to the manufacturer’s procedures.

### siRNAs

ON-TARGET plus non-targeting Control (D-001810-01, D-001810-02, D-001810-03, D-001810-04) siRNAs and FGFR4-targeting (J-003134-12, J-003134-13, J-003134-14, J-003134-15) siRNA were purchased from Dharmacon.

### Cell lines and transfection

The RMS559 cell line was established in Prof. Jonathan Fletcher’s laboratory, and the RH30 cell line was a generous gift from Prof. Ola Myklebost. The RD (CCL-136) cell line was obtained from ATCC. U2OS cells stably expressing wild-type FGFR4 (U2OS-FGFR4) have been described previously [22]. RMS559 cells were propagated in Iscove’s Modified Dulbecco medium supplemented with 15% foetal bovine serum and GlutamaxTM. RH30 cells were kept in RPMI media supplemented with 10% foetal bovine serum, and the RD cells and the U2OS-FGFR4 cells were cultured in DMEM media with 10% foetal bovine serum. All cell lines were grown in a 5% CO_2_ atmosphere at 37 °C. The cell lines were tested negative for mycoplasma contamination by PCR. siRNA-mediated knockdown was performed using Lipofectamine RNAiMAX transfection reagent (Thermo Fisher Scientific) according to the manufacturer’s protocol. In total, 10 nM of siRNA concentration was used and experiments were performed 48–96 h after transfection.

### Sequencing

Whole-exome sequencing was performed by the Oslo University Hospital Genomics Core Facility (oslo.genomics.no) using the TWIST Bioscience Human Core Exome Kit with additional RefSeq content. The sequencing library was prepared following the manufacturer’s protocols starting from 50 ng of genomic DNA and sequenced 2 × 76 bp on an Illumina NextSeq 500 instrument. Fastq files were uploaded into Illumina’s Sequence Hub, and single nucleotide variants and short insertions/deletions were identified using the DRAGEN Somatic Pipeline v.3.4.5 with hg38 as the human genome assembly reference. All variants were further annotated with the Variant Effect Predictor (v100), using GENCODE (v33) as the transcript reference [23]. To highlight variants in cancer-relevant genes, we annotated genes with data from COSMIC’s Cancer Gene Census (v91), as well as status with respect to tumour suppressor genes/oncogenes, the latter harvested from CancerMine (v23) and Network of Cancer Genes (v6.0), using the built-in data bundle from the Personal Cancer Genome Reporter [24–27].

Since exome sequencing of cancer cell lines without matching normal samples is bound to generate a mix of germline variants and somatic variants, we removed variants that overlapped with germline variants found in gnomAD (release 2.1), specifically those with minor allele frequency >0.2% in any population [28, 29]. We further restricted the filtered variant set to coding variants only (missense, stopgain/stoploss, frameshift/non-frameshift, splice site donor/acceptor).

Whole transcriptome mRNA-sequencing was performed by the Oslo University Hospital Genomics Core Facility using the Illumina TruSeq Stranded mRNA Kit following Illumina’s protocol. The RNA libraries were sequenced 2 × 75 bp on an Illumina NextSeq 500 instrument. The fastq files were mapped to the hg38 human genome reference and analysed using the DRAGEN RNA Pipeline v.3.4.5 in Sequence Hub. Transcript Per Million (TPM) was calculated for every gene and used as the value of gene expression.

### Western blot

Upon indicated treatment, cells were washed in cold PBS and lysed in lysis buffer (0.1 M NaCl, 10 mM Na2PO4, 1% Triton X-100 and 1 mM EDTA, pH 7.4) supplemented with protease and phosphatase inhibitors at 4 °C. The lysate was mixed with 4× sample buffer (Bio-Rad). In some cases, the cells were lysed directly in the sample buffer. Lysates were subjected to sodium dodecyl sulphate-polyacrylamide gel electrophoresis (SDS-PAGE) using 4–12% gradient gels (Bio-Rad) followed by blotting onto a PVDF membrane (Bio-Rad) using the TransBlot^®^ Turbo Transfer system (Bio-Rad). Membranes were then incubated with indicated primary antibodies followed by corresponding secondary antibodies coupled to HRP. Bands were visualised by chemiluminescence using SuperSignal West Dura Extended Duration Substrate (Thermo Fisher Scientific) or SuperSignal West Femto Maximum Sensitivity Substrate (Thermo Fisher Scientific) and detected using ChemiDoc Imaging System (Bio-Rad). In some cases, antibodies were stripped from the membranes using Restore Western Blot Stripping buffer (Thermo Fisher Scientific), and the membranes were reprobed. The images were prepared using ImageLab Software (Bio-Rad) and Adobe Illustrator CS4 14.0.0 (San Jose, CA, USA).

### IncuCyte cell growth analysis

RMS559 cells were seeded on 96-well plates, six wells for each condition. The next day, the cells were transfected with scramble or FGFR4 targeting siRNAs. After 16 h, the media were changed, and cells were kept for 6 days in media containing 1.5% serum. Cells were imaged every 3 h, and cell confluency was quantified by Essen Bioscience InCucyte FLR. At least three wells with similar starting confluency were selected for growth curves.

### MTS assay

RMS559 cells were seeded on 96-well plates. The next day, the cells were treated with siRNAs or increasing concentrations of inhibitors for 72 h or 6 days. Cell viability was determined using MTS assay (Promega), according to the manufacturer’s protocol. The absorbance at 490 nm was measured using Victor X3 multiplate reader (PerkinElmer), and the absorbance values were corrected by blank sample measurements (without cells) and normalised to DMSO control (when inhibitors were used) or the mean of all samples within an experiment (when siRNAs were used). Three replicates for all conditions were performed for all experiments. Data were processed in GraphPad Prism 8 (GraphPad Software, La Jolla, CA, USA). IC_50_ values were calculated from a log([inhibitor]) versus normalised response curve fit using GraphPad Prism 8.

### Light microscopy

Cells seeded on coverslips were treated as indicated and fixed in ~4% formaldehyde (Sigma). Next, the cells were permeabilized in 0.1% Triton X-100 and stained with indicated antibodies and Hoechst 33342 (Thermo Fisher Scientific). The coverslips were mounted in ProLong Diamond Antifade Mounting (Thermo Fisher Scientific) reagent and examined with a ×63 objective on a Zeiss confocal Laser Scanning Microscope (LSM) 880 (Jena, Germany). Images were prepared with Fiji Image J software and Adobe Illustrator CS4 14.0.0.

### Immunoprecipitation

RMS559 cells treated as indicated were lysed in lysis buffer (20 mM Tris-HCL pH 7.4, 150 mM NaCl, 1 mM EDTA, 1% Triton X-100 supplemented with protease and phosphatase inhibitor cocktail). A small fraction of the lysate was kept for analysis of proteins in the input while the rest of the lysate was incubated for 1 h at 4 °C with the indicated antibodies. One sample was kept with normal rabbit IgG as a negative control. After the incubation, Protein G Sepharose beads were added to each sample, and the incubation at 4 °C was continued for another 1 h. Finally, the Sepharose pellets in all samples were washed three times with lysis buffer and subjected to SDS-PAGE followed by western blotting using denoted antibodies.

### Phosphoproteomics

#### Sample preparation

Cells treated with LY2874455 or FGF1 were lysed in RIPA buffer and homogenised with a sonicator (30 s × three times with 30-s interval). Insoluble material was removed by centrifugation. Protein concentrations were estimated by BCA assay (Pierce). For each replicate, an equal amount (600 µg) of protein was precipitated on amine beads as previously described [30]. The precipitated proteins on beads were dissolved in 50 mM ammonium bicarbonate, reduced, alkylated and digested with trypsin (1:50 enzyme:protein ratio; Promega) at 37 °C overnight. Digested peptides were transferred to a new tube, acidified, and the peptides were de-salted using Oasis cartridges for STY peptide enrichments. Phosphorylated peptides were enriched using TiO_2_ -IMAC magnetic beads. Enriched peptides were de-salted by C18-stage tips.

#### LC-MS/MS

Peptide samples were dissolved in 10 µl 0.1% formic buffer and 3 µl loaded for MS analysis. LC-MS/MS analysis of the resulting peptides was performed using an Easy nLC1000 liquid chromatography system (Thermo Electron, Bremen, Germany) coupled to a QExactive HF Hybrid Quadrupole-Orbitrap mass spectrometer (Thermo Electron) with a nanoelectrospray ion source (EasySpray, Thermo Electron). The LC separation of peptides was performed using an EasySpray C18 analytical column (2 µm particle size, 100 Å, 75-μm inner diameter and 25 cm; Thermo Fisher Scientific). Peptides were separated over a 120 min gradient from 2% to 30% (v/v) acetonitrile (ACN) in 0.1% (v/v) formic acid (FA), after which the column was washed using 90% (v/v) ACN in 0.1% (v/v) FA for 20 min (flow rate 0.3 μL/min). All LC-MS/MS analyses were operated in a data-dependent mode where the most intense peptides were automatically selected for fragmentation by high-energy collision-induced dissociation.

#### Data analysis

Raw files from the LC-MS/MS analyses were submitted to MaxQuant (v1.6.17.0) software for peptide/protein identification [31]. The following parameters were set: Carbamidomethyl (C) was set as a fixed modification; protein N-acetylation and methionine oxidation as variable modifications and PTY. A first search error window of 20 ppm and main search error of 6 ppm was used. Minimal unique peptides were set to one, and FDR allowed was 0.01 (1%) for peptide and protein identification. The Uniprot human database was used. Generation of reversed sequences was selected to assign FDR rates. MaxQuant output files (STY(sites).txt were uploaded to the Perseus software. Identifications from potential contaminants and reversed sequences were removed, and intensities were transformed to log2. Identified phosphorylation sites were filtered only for those that were confidently localised (class I, localisation probability ≥0.75). Next, proteins identified in two out of three replicates were considered for further analysis. All zero intensity values were replaced using noise values of the normal distribution of each sample. Protein or STY abundances were compared using LFQ intensity values and a two-sample Student’s *T* test (permutation-based FDR correction (250 randomisations), FDR cut-off: 0.05, S0: 0.1).

### Animal experiments

#### Animal information

For all experiments, female HSD:Athymic Nude Foxn1nu were used. The mice were locally bred at the Department of Comparative Medicine at the Norwegian Radium Hospital, Oslo, Norway. The mice were housed under pathogen-free conditions in Eurostandard III cages with two to eight mice in each cage. The cages were routinely changed one time per week. The mice had ad libitum access to nr. 3 breeding pellets (Special Diet Services) and water from bottles acidified to pH 3. For environmental enrichment, the mice had access to nesting paper and plastic houses. The environmental conditions in the animal rooms were as follows; 62% (±5%) relative humidity, air change cycles of 15 h, a temperature of 22 °C (±1 °C) and a light cycle of 12 h (1 lux at night, 70 lux at day).

#### In vivo evaluation of phosphorylation levels after inhibitor treatment

Six female athymic nude mice were injected s.c. with 1 × 10^6^ RMS559 cells in serum-free medium on the right and left flank. The tumours were allowed to grow to ~300–400 mm^3^ in size before the mice were treated orally with 10 mg/kg FGF401 or vehicle (30% PEG300, 5% Tween80 and 22% DMSO). The mice were sacrificed at 3, 6, 16 and 24 h after treatment. In the case of LY2874455 mice were treated orally with 6 mg/kg of the inhibitor or vehicle (30% PEG300, 5% Tween80 and 2% DMSO). The mice were sacrificed at 3 or 6 h after treatment. The tumours were snap-frozen in liquid nitrogen. Proteins were isolated from the samples using Precellys Evolution Homogenizer (Bertin instrument, Montigny-le-Bretonneux, France). Lysates were subjected to western blot analysis.

#### Treatment efficacy

Five- to six-week-old female athymic nude mice were injected s.c. with 1 × 10^6^ RMS559 cells in serum-free medium on the right and left flank. After about 3 weeks, the mice were divided into two treatment groups of six and seven mice. Each group consisted of ten tumours with an average tumour volume of about 60 mm^3^. The mice were given 10 mg/kg FGF401 or vehicle (30% PEG300, 5% Tween80 and 22% DMSO) in a volume of 10 µl/g body weight orally. In the case of LY2874455, the mice were given 6 mg/kg inhibitor or vehicle (30% PEG300, 5% Tween80 and 2% DMSO). Both vehicle and inhibitors were given twice daily (at 6 h of intervals) on weekdays, and once during the weekends for a total of 25 treatments. Treatment toxicity was monitored by daily weight loss measurements and body conditioning assessments. Tumour volumes were followed two times per week by measuring tumour diameter with a calliper. The tumour volumes were calculated by the formula 0.5 × length × width^2^. The experiment was terminated when the average tumour volume of the mice receiving the vehicle reached 1000 mm^3^. At the end of the experiment, the tumours were collected, weighed and snap-frozen in liquid nitrogen. The growth data are presented as average tumour volume (mm^3^) ± SEM.

### Statistical analysis

The sample size estimation for the animal experiments was based on our previous power analyses and experience using mice models, as well as previous publications [19, 32]. All other experiments that were further subjected to statistical analysis or IC_50_ estimation were performed with the use of three independent experiments unless noted in the figure legends, as that sample size is generally accepted in the field. Variation in the data was presented using standard deviation (SD), standard error of the mean (SEM) or confidence intervals (CI), as described in the figure legends. Statistical analysis of the proteomic data was performed as described previously in “Materials and methods”. All other statistical analysis was performed using GraphPad Prism 8.4.3 with the use of appropriate statistical tests as indicated in the figure legends. Normal distribution and equal variance between the compared groups were assumed, unless indicated otherwise. Time-course cell proliferation was analysed by linear regression, and the slopes were compared using software build-in test, calculating the *P* value (two-tailed) by testing against the null hypothesis that the slopes are identical (the lines are parallel).

## Results

### Genomic characterisation of RMS559

**Fig. 1:**
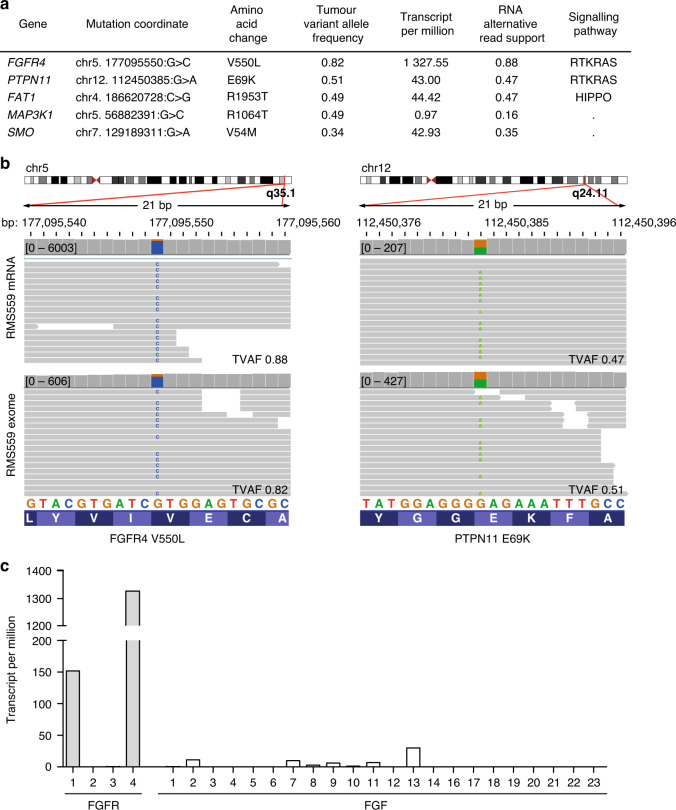
Genomic characterisation of RMS559 cells. **a** An overview of mutations identified in RMS559 cells. **b** Graphics from Integrative Genomic Viewer. Sequencing reads mapping to different segments of the human genome, *FGFR4* and *PTPN11* genes. TVAF tumour variant allele fraction. **c** Expression levels of FGF family members in RMS559 cells. Note FGF11-14 are not secreted and are thus not activating FGFRs.

To investigate strategies to inhibit FGFR4-dependent rhabdomyosarcoma growth we searched for a model cell line suitable for this purpose. The RMS559 rhabdomyosarcoma cell line was established from an embryonal rhabdomyosarcoma arising in a 5-year-old patient. RMS559 cells were previously suggested to harbour the FGFR4 V550L mutation [33]. To validate this and to determine the expression of the mutated allele, we performed both exome and RNA sequencing. The FGFR4 V550L mutation was identified at both the DNA and RNA levels with a high allelic fraction (0.82 and 0.88, respectively) (Fig. 1a, b). The phosphatase PTPN11 (protein tyrosine phosphatase non-receptor type 11), also known as SHP2 (Src homology region 2 domain-containing phosphatase 2), was also mutated at a high allelic fraction (0.51) (Fig. 1a, b). Interestingly, PTPN11 is involved in the regulation of the RAS/MAPK pathway downstream of receptor tyrosine kinases, including FGFR4 [34]. The identified E69K mutation is recurrent according to COSMIC [35] (cancer.sanger.ac.uk), and is predicted to activate PTPN11 [34]. Apart from these two recurrent hotspot mutations, few oncogenic mutations were found in RMS559 when we examined driver oncogenes and tumour suppressor genes defined by CancerMine [24]. The identified FAT1 R1953T, MAP3K1 R1064T and SMO V54M mutations are not recurrent according to COSMIC and have not previously been shown to be involved in oncogenesis [35]. Our data are in line with previous studies showing that RMS harbours in general few mutations, reflecting their origin in young children [7].

Transcriptome sequencing revealed that FGFR4 is highly expressed in RMS559 cells (Fig. 1c), and that FGFR1 is moderately expressed. Other FGFRs were expressed at very low levels. Among the FGFs, FGF13, an intracellular FGF incapable of activating FGFRs, is the most expressed ligand. The other FGFs show very low expression levels. It is therefore unlikely that an autocrine FGF/FGFR stimulation loop plays any major role in these cells. Furthermore, relevant downstream adaptors and effectors of FGFR4, including PTPN11, are also expressed by RMS559 cells (Supplementary Fig. 1). PTPN11 (TPM = 43.0) seem to be expressed in a typical range for human cancer cells (20–50) [36].

### RMS559 growth is dependent on FGFR4 signalling

The FGFR4 V550L mutation identified in RMS559 cells is located close to the active site of the tyrosine kinase domain and occludes the binding of drugs that target the active site. The mutation is, therefore, a so-called “gate-keeper” mutation [37]. In addition, this mutation autoactivates the receptor causing ligand-independent signalling [37].

**Fig. 2:**
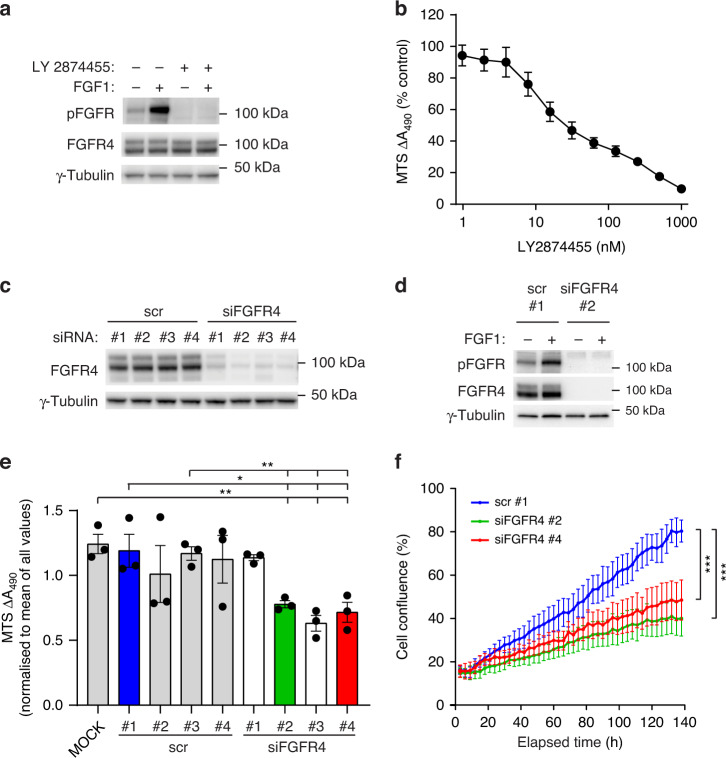
Characterisation of FGFR activity in RMS559 cells. **a** Analysis of FGFR4 activation in RMS559 cells. RMS559 cells were serum-starved and treated with 100 ng/ml FGF1 and 10 U/ml heparin for 20 min in the presence or absence of the FGFR inhibitor LY2874455 (100 nM, 1 h). Cells were then lysed, and the lysates were analysed by western blotting using the indicated antibodies. One representative experiment out of at least three independent experiments is presented. **b** Dependency of RMS559 on FGFR signalling for viability. RMS559 cells were treated with increasing concentrations of FGFR inhibitor (LY2874455) for 72 h before measurement of cell viability using an MTS assay. Data were normalised to the DMSO control and presented as means ± SEM of three independent experiments. **c** Knockdown efficiency of FGFR4 in RMS559 cells. RMS559 cells were treated with FGFR4 siRNAs (#1-#4) or non-coding, control siRNA (scr #1-#4) for 72 h. Cells were then lysed, and the lysates were analysed by western blotting using the indicated antibodies. **d** Signalling upon FGFR4 knockdown. RMS559 cells were treated with FGFR4 siRNA #2 or non-coding, control siRNA (scr #1) for 48 h. Cells were then serum-starved and treated with 100 ng/ml FGF1 and 10 U/ml heparin for 15 min. Cells were then lysed and the lysates were analysed by western blotting using the indicated antibodies. One representative experiment out of at least two independent experiments is presented. **e** Viability of RMS559 cells upon FGFR4 knockdown. RMS559 cells were transfected as indicated (MOCK-transfection reagent only). The cells were kept in 1.5% serum media for 72 h before measurement of cell viability using MTS assay. Data were normalised to the mean of all values obtained in each experiment and presented as means ± SEM of three independent experiments. The effect of FGFR4 knockdown against controls were analysed using two-tailed unpaired *t* tests, **P < 0.01, *P < 0.05, *n* = 3. **f** Proliferation of RMS559 cells upon FGFR4 knockdown. RMS559 cells were transfected as indicated and kept in 1.5% serum media. Cell confluence was measured every third hour for 140 h using Essen Bioscience Incucyte FLR. The growth curves represent means ± SEM of three independent experiments. Linear regression analysis was used and the slopes were tested for significant difference ***P < 0.001, *n* = 3.

We, therefore, decided to investigate the activity of FGFR4 in RMS559 cells using phospho-specific antibodies against the activated forms of FGFRs. We observed by western blotting activated FGFR4 in the absence of FGF stimulation (Fig. 2a). This signal was increased by the addition of FGF1, indicating that there is a potential for increased signalling by ligand stimulation in these cells. The inhibitor LY2874455 has previously been shown to inhibit all FGFRs, including FGFR4 with gatekeeper mutations [32, 38]. We applied LY2874455 to RMS559 cells and observed complete inhibition of activated FGFR4, demonstrating the efficacy of the drug against FGFR4 V550L activity in this cell line (Fig. 2a).

Next, to test whether RMS559 growth is dependent on FGFR signalling, we performed MTS viability assays with increasing concentrations of LY2874455 (Fig. 2b). LY2874455 strongly inhibited the viability of RMS559 cells, indicating that their growth is dependent on FGFR signalling. As LY2874455 inhibits all four FGFRs, we used siRNAs to knock down FGFR4 specifically. Four different siRNAs, which all knocked down FGFR4 although at varying degrees, were tested (Fig. 2c). First, we investigated signalling upon FGFR4 knockdown by stimulating RMS559 cells with FGF1 (Fig. 2d). Although RMS559 cells also express some FGFR1 and our antibody detecting phosphorylated FGFR is not specific for FGFR4, we did not observe much active FGFR when FGFR4 was knocked down. This indicates that FGFR4 is the main signalling receptor in RMS559 cells. In MTS cell viability assays, RMS559 cells treated with FGFR4 siRNA oligos #2, #3 and #4 showed a clear reduction in viability, meanwhile oligo #1 had no effect (Fig. 2e). These results correlate with knockdown efficiencies of FGFR4 as siRNA oligo #1 was the least efficient siRNA oligo. We also evaluated the role of FGFR4 signalling in proliferation by live-cell imaging. Using two siRNAs against FGFR4, we observed a strong reduction in proliferation (Fig. 2f). Taken together, the data indicate that RMS559 cells are oncogenically dependent on FGFR4 signalling.

### Characterisation of FGFR4 localisation in RMS559

**Fig. 3:**
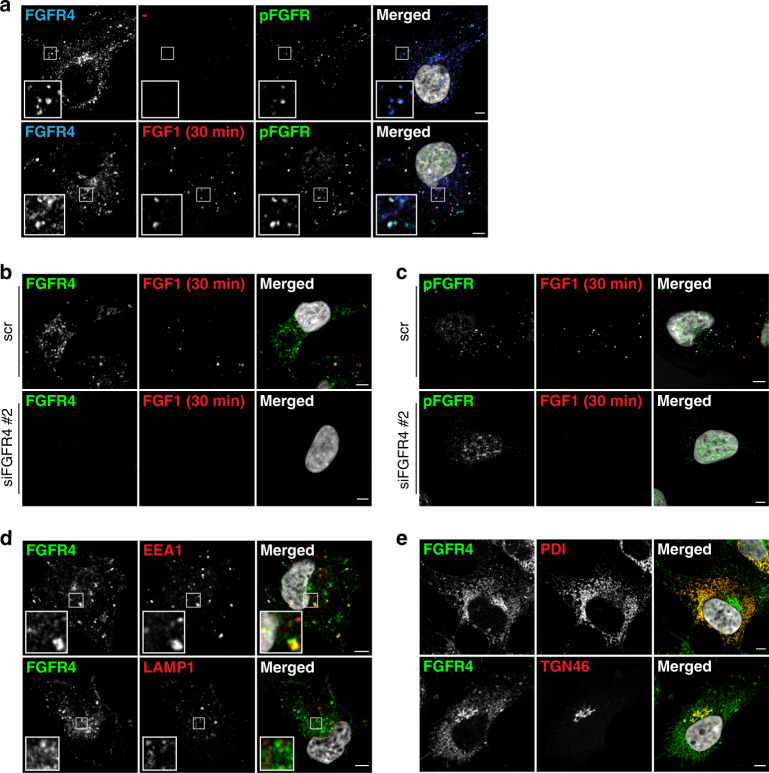
Intracellular localisation of FGFR4 in RMS559 cells. **a** RMS559 cells growing on coverslips were left untreated (−) or incubated with DL550-FGF1 (100 ng/ml) and heparin (50 U/ml) for 30 min. The cells were then fixed and stained with anti-FGFR4 and anti-pFGFR (phospho-FGFR) antibodies and Hoechst and analysed by confocal microscopy. Images were taken at fixed intensity settings for DL550-FGF1, and brightness/contrast was adjusted equally in all images for DL550-FGF1. Scale bar, 5 µm. Inserts 3× enlarged. **b**, **c** RMS559 cells growing on coverslips were transfected with non-coding, control siRNAs (scr) or siRNAs targeting FGFR4 (siR4 #2) for 48 h. Then the cells were left untreated (−) or incubated with DL550-FGF1 (FGF1) and heparin (50 U/ml) for 30 min. The cells were then fixed and stained with anti-FGFR4 (**b**) or anti-pFGFR (**c**) antibodies and Hoechst and analysed by confocal microscopy. Images were taken at fixed intensity settings, and brightness/contrast was adjusted equally for all images. Scale bar, 5 µm. Images with lower magnification from this experiment are shown in Supplementary Fig. 3. **d** RMS559 cells growing on coverslips were fixed and stained with anti-FGFR4 and anti-EEA1 (upper panel) or anti-LAMP1 (lower panel) antibodies and Hoechst. The cells were then analysed by confocal microscopy. Scale bar, 5 µm. Inserts 3× enlarged. **e** RMS559 cells growing on coverslips were fixed and stained with anti-FGFR4 and anti-PDI (upper panel) or anti-TGN46 (lower panel) antibodies and Hoechst. The cells were then analysed by confocal microscopy. Scale bar, 5 µm.

To explore the intracellular localisation of FGFR4 in RMS559 cells, we turned to confocal microscopy using FGFR4 antibodies. We observed that FGFR4 was mainly found in intracellular structures (Fig. 3a and Supplementary Fig. 2a). This does not exclude that the FGFR4 V550L is present at the cell surface, as the fluorescent signal from the cell surface is often much weaker due to its spread over a larger area than a signal accumulating in smaller intracellular compartments. On the contrary, since external addition of FGF1 clearly increased FGFR4 activation (Fig. 2a, d), we suspect that a substantial portion of FGFR4 is localised at the cell surface in these cells.

We also probed for active receptors, using phospho-FGFR (pFGFR) antibodies, and observed staining for pFGFR both in the absence and in the presence of FGF1 (Fig. 3a, upper and lower panel, respectively, and Supplementary Fig. 2b). Upon addition of FGF1 the staining for pFGFR was somewhat increased. This is in accordance with the western blot findings. FGFR4 V550L seems to be constitutively active but can be further activated by the addition of a ligand. We also observed that FGF1 was present in intracellular structures (Fig. 3a, lower panel) indicating that externally added FGF1 is taken up by the cells by endocytosis. This also supports the idea that FGFR4 is present at the cell surface.

Moreover, substantial overlap between anti-FGFR4 and anti-pFGFR staining (both with and without ligand) was observed, indicating specific staining. To confirm this, we also stained cells after siRNA-mediated FGFR4 knockdown. Little FGFR4 antibody staining was observed when FGFR4 was knocked down (Fig. 3b and Supplementary Fig. 2a). Nuclear staining was observed in the case of pFGFR even in the FGFR4 knockdown cells, indicating some nonspecific staining (Fig. 3c and Supplementary Fig. 2b). Moreover, FGF1 was not observed in cells upon FGFR4 knockdown. This supports that externally added FGF1 is taken up into the cells by surface-localised FGFR4.

Next, we wanted to investigate which intracellular structures FGFR4 localised to. For this purpose, we used antibodies against known intracellular marker proteins and investigated the colocalization between FGFR4 and the actual marker. As can be seen in Fig. 3d, FGFR4 colocalized with markers of the endosomal pathway, both with the early endosomal marker, EEA1 (upper panel), and the late endosome/lysosome marker, LAMP1 (lower panel). FGFR4 was also shown to localise extensively to the secretory pathway (PDI: endoplasmic reticulum (ER), upper panel and TGN46: Trans-Golgi network, lower panel) (Fig. 3e).

Taken together, the data indicate that FGFR4 V550L is present both in the secretory and the endocytic pathway, as well as at the cell surface. The latter could be important for therapeutic purposes as the receptor must be exposed to the extracellular environment to bind to therapeutic antibodies or antibody–drug conjugates.

### Targeting downstream FGFR4 V550L signalling

**Fig. 4:**
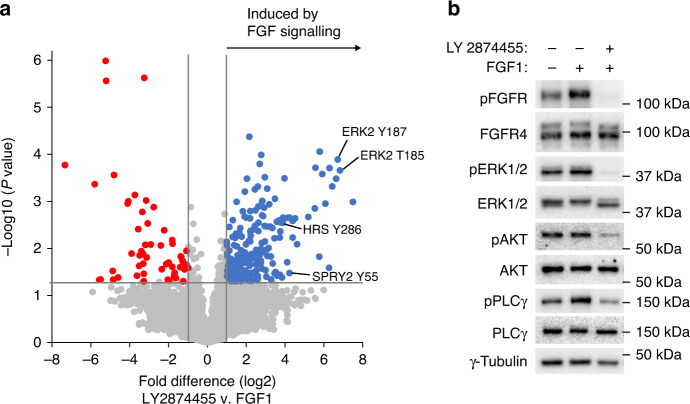
Signalling pathways downstream of FGFR4 V550L. **a** Phosphoproteomic analysis of FGFR4 signalling in RMS559. Lysates from RMS559 cells treated with FGF1 (100 ng/ml) or LY2874455 (100 nM) were subjected to phosphoproteomics as described in materials and methods. Three replicates for each condition were performed. **b** Western blot analysis of FGFR downstream signalling. RMS559 cells were stimulated as indicated before lysis. The lysates were subjected to SDS-PAGE and western blotting using denoted antibodies. One representative experiment of at least two independent experiments is presented.

A possible strategy to inhibit oncogenic signalling from mutant FGFR4 could be to target its downstream signalling pathways. To explore the phosphorylation network initiated by FGFRs in RMS559 cells, we used phosphoproteomics. RMS559 cells treated with FGF1 (to boost FGFR activation) or with LY2874455 (to inactivate FGFR signalling) were subjected to phosphoproteomic analysis (Supplementary Fig. 3a). The cells were lysed, and phosphopeptides were enriched using TiO_2_ beads before being analysed by label-free quantitative mass spectrometry. In total, we identified 6225 phosphopeptides. We defined phosphopeptides to be dependent on FGFR signalling if they were significantly downregulated more than twofold (*P* < 0.05) when treated with LY2874455. By this method, we identified 207 peptides to be upregulated, and 57 to be downregulated potentially by FGFR kinase signalling (Fig. 4a). Reassuringly, tyrosine phosphorylated peptides in the activation loop of FGFR4 were identified, as well as many peptides mapping to signalling pathways often associated with RTK signalling (Table 1 and Supplementary Table 1).

**Table 1:** Tyrosine phosphorylation sites downstream of FGFR4 V550L in RMS559 cells identified by phosphoproteomics.

Protein name	Protein activity	Site	Upstream kinase	RTK induced*	FGFR induced*	Fold change
ERK2	Kinase	Y187	MEK1	+	+	105
Sprouty 2	Negative regulator	Y55	Src	+	+	19
DDR2	RTK	Y481		+		18
HRS	Endosomal transport	Y286		+		14
CTNND1	Adherens junctions stabilisation	Y904	CSFR	+	+	13
N-WASP	Actin polymerisation	Y256	Arg	+	+	11
Sprouty 1	Negative regulator	Y53		+	+	9
Ack	Kinase	Y827		+		5
Sprouty 4	Negative regulator	Y52		+		4
JAM3	Tight junctions	Y293				3
MEGF10	Complement component	Y1061			+	3
EGFR	RTK	Y1197	EGFR	+		3
Latrophilin 2	GPCR	Y1406		+		3
FGFR4	RTK	Y642	FGFR4	+	+	2
VANGL2	Adaptor	Y308				2
Sprouty 2	Negative regulator	Y176			+	2

*RTK* receptor tyrosine kinase, *GPCR* G-protein coupled receptor.

The information regarding the phosphorylation sites was found on www.phosphosite.org.

*Phosphorylation induced or repressed by stimulation or inhibition, respectively of RTK/FGFR pathway.

Then, we used the Ingenuity Pathway Analysis (IPA) software to identify enriched canonical pathways in the dataset predicted to be dependent on FGFR signalling (Supplementary Fig 3b).

As expected, receptor tyrosine kinase signalling, including FGF signalling, was among the significant pathways, in addition to MAPK, PI3K/mTOR and PLCγ/PKC pathways. We confirmed by western blotting that these signalling pathways were activated by FGFRs in RMS559 cells (Fig. 4b). Interestingly, a previous functional screen for inhibitors of mutant FGFR4 revealed that PI3K/mTOR inhibitors had a strong anti-proliferative effect [14].

The phosphoproteomic analysis of RMS559 (Fig. 4a, Table 1, Supplementary Table 1 and Supplementary Fig. 2) and the western blotting (Fig. 4b) revealed signalling pathways that potentially can be targeted. For the RAS/MAPK and the PI3K/AKT pathways, several inhibitors are showing promising results in clinical trials. We, therefore, tested the effect of inhibiting these two pathways on RMS559 cell viability.

**Fig. 5:**
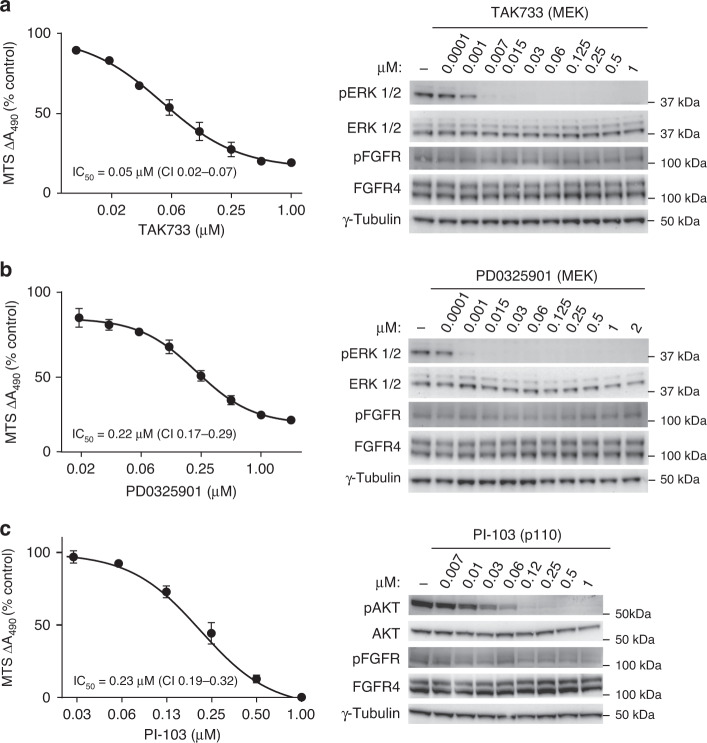
Efficacy of RAS/MAPK and PI3K/AKT pathway inhibitors in RMS559 cells. The efficiency of the RAS/MAPK and PI3K/AKT pathway inhibitors TAK733 (**a**), PD0325901 (**b**) and PI-103 (**c**) in RMS559 cells were examined by cell viability MTS assays (left) and western blots (right). For MTS assays, RMS559 cells were treated with increasing concentrations of the inhibitors (as indicated) for 6 days before measurement of cell viability using the MTS assay. Data were normalised to the DMSO control and presented as means ± SEM of three independent experiments (except TAK733 0.007 µM and PD0325901 0.015 µM and 0.03 µM, which were performed twice). Curves were fitted with non-linear regression (Hill equation with variable slope), IC_50_ ± 95% CI (Confidence Interval levels). Note the different scales on the *x* axes (log2). For western blotting, RMS559 cells were kept in serum-free media for 1 h prior to 1 h of incubation with DMSO (−) or increasing concentrations of the inhibitors. Cells were then lysed, and the lysates were analysed by western blotting using the indicated antibodies. One representative experiment of at least two for each inhibitor is shown.

To test the importance of the RAS/MAPK pathway on FGFR4 V550L-driven cell viability, inhibitors targeting either Raf or MEK1/2 were used (Fig. 5a, b and Supplementary Fig. 4). The efficiency of the inhibitors was confirmed by western blotting using antibodies to detect phosphorylated ERK 1/2 (pERK 1/2). All the inhibitors tested reduced phosphorylation of ERK 1/2 accompanied by a clear reduction in RMS559 cell viability, measured by the MTS assay. The inhibitors showed, however, quite different efficiency. We observed that both the pan-RAF (TAK632) and the RAF/MEK (RO51267663) (Supplementary Fig. 4a, b) inhibitors were less efficient than the MEK inhibitors, TAK733 and PD0325901 (Fig. 5a, b). TAK733 and PD0325901 efficiently inactivated the pathway at nanomolar concentrations, while TAK632 and RO51267663 were slightly less efficient. The MEK1/2 inhibitor, UO126 was the least efficient inhibitor, requiring concentrations in the µM range to inactivate the pathway (Supplementary Fig. 4c). The inhibitors’ effect on RMS559 cell viability correlated with their ability to efficiently downregulate the signalling pathway.

In order to test the importance of PI3K/AKT signalling pathway on FGFR4 V550L-driven cell viability, an inhibitor targeting p110 (PI-103), a subunit of PI3K, was used. PI-103 efficiently inhibited PI3K activity as measured by phospho-AKT antibodies (Fig. 5c). The viability of RMS559 cells measured by the MTS assay corresponded well with PI-103s inhibitory activity on PI3K signalling. We also tested the PI3K inhibitor LY294002, which also reduced both FGFR signalling activity and viability (Supplementary Fig. 5a). Although the efficiency of the two inhibitors varied, a reduction in RMS559 cell viability was observed in both cases (Fig. 5c and Supplementary Fig. 5a).

Taken together, both the RAS/MAPK and the PI3K/AKT signalling pathways contribute to RMS559 cell viability.

Interestingly, the phosphatase PTPN11 was found to be mutated (E69K) in the cell line. The PTPN11 E69K occurs in the inhibitory SH2-domain and is predicted to activate PTPN11 leading to increased MAPK signalling [34]. The PTPN11 inhibitor, SHP099, has been reported to efficiently inhibit PTPN11 E69K [34]. However, treating RMS559 cells with SHP099 gave little effect on cell viability, although similar concentrations of SHP099 lead to some decrease in phosphorylated ERK 1/2, a downstream target of PTPN11 (Supplementary Fig. 5b). The weak effect on proliferation indicates that PTPN11 E69K is not a major driving force in these cells and that a stronger inhibitory effect on ERK 1/2 phosphorylation is necessary to reduce viability. To confirm the efficiency of SHP099 towards the mutant PTPN11, we also tested SHP099 in U2OS cells with endogenous wild-type PTPN11 and stably expressing wild-type FGFR4 (U2OS-FGFR4). In this cell line, SHP099 reduced FGF1-induced ERK activation as efficiently as in the RMS559 cells (Supplementary Fig. 5c). This indicates that the inhibitor is similarly efficient towards inhibiting wild-type PTPN11 and PTPN11 E69K, as previously reported [34]. Although PTPN11 E69K is constitutively active in RMS559 cells, it is probably not a major oncogenic event, pointing to FGFR4 V550L as the main driving force in these cells.

### Targeting FGFR4 in rhabdomyosarcoma with HSP90 inhibition

**Fig. 6:**
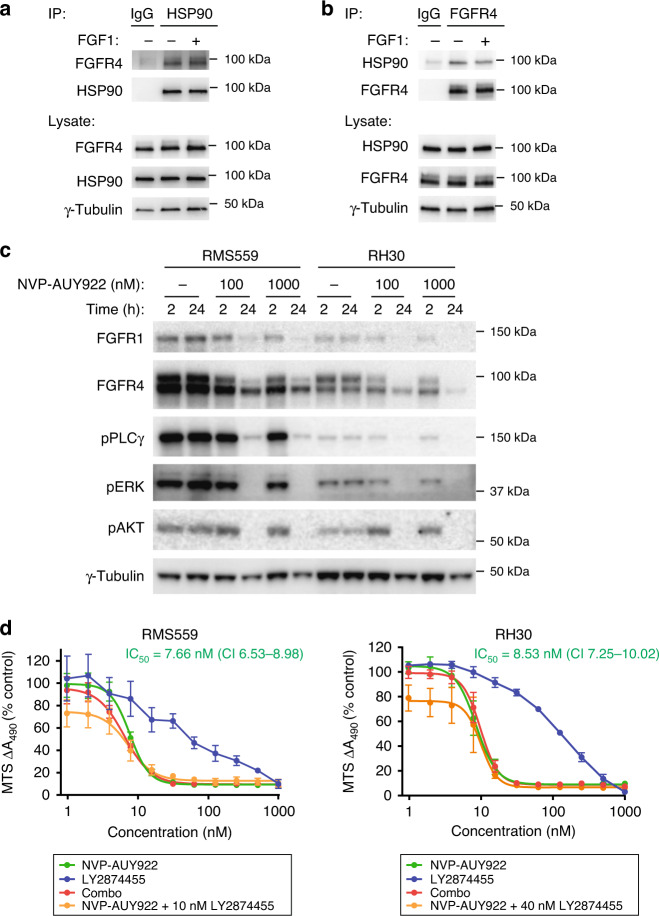
FGFR4 V550L is dependent on HSP90 activity. RMS559 cells were treated with FGF1 as indicated, lysed and the lysates subjected to immunoprecipitations using anti-HSP90 (**a**), or anti-FGFR4 (**b**) antibodies and analysed by western blotting. Normal rabbit IgG was included as the control. **c** RMS559 and RH30 cells were treated with NVP-AUY922 to block HSP90 activity for the indicated times, lysed, and the lysates were analysed by western blotting using denoted antibodies. One representative experiment of three independent experiments is shown. **d** RMS559 and RH30 cells were treated with indicated inhibitors for 72 h before cell viability was evaluated using MTS assay. Combo represents both inhibitors. Data were normalised to the DMSO control and presented as mean ± SEM of three independent experiments. Curves for NVP-AUY922, Combo and NVP-AUY922 with fixed concentration of LY2874455 were fitted using Hill equation with variable slope, IC_50_ ± 95% CI (confidence interval levels).

As FGFR4 seems to accumulate in the secretory pathway (Fig. 3d), we speculated that the folding of FGFR4 V550L protein in RMS559 cells could be compromised and possibly depend on heat shock protein 90 (HSP90). Therefore, as an alternative targeting strategy, we tested whether the mutant FGFR4 was dependent on HSP90 for its activity. First, we checked the potential binding between FGFR4 V550L and HSP90 by immunoprecipitation using antibodies against HSP90 (Fig. 6a) or antibodies against FGFR4 (Fig. 6b). Both approaches confirmed binding between HSP90 and FGFR4 V550L indicating that the latter might be a client protein of HSP90. We then investigated if inhibition of HSP90 activity could affect the protein level of mutant FGFR4 V550L in RMS559 cells. We also included the rhabdomyosarcoma cell line, RH30, expressing wild-type FGFR4 in these experiments. Upon treatment with an HSP90 inhibitor, NVP-AUY922, for 2 h, FGFR4 V550L levels in RMS559 cells were reduced, and a further reduction was observed after 24 h of treatment (Fig. 6c). A similar pattern was observed upon treatment of RH30 cells, suggesting that the HSP90 dependency is not due to the V550L mutation. We were also able to detect FGFR1 in both RMS559 and RH30 cell lysates, and the corresponding band was also reduced upon HSP90 inhibition (Fig. 6c). In both cell lines, the FGFR-related signalling pathways were also decreased along with the disappearance of FGFR4 upon HSP90 inhibition. Thus, our results suggest a potential therapeutic opportunity for FGFR targeting with HSP90 inhibitors.

We also tested the stability of FGFR4 in RMS559 and RH30 cells upon a wider concentration range of NVP-AUY922 (Supplementary Fig. 6). The HSP90 inhibitor seemed to severely downregulate FGFR4 protein levels (and downstream signalling) at low concentrations and showed a steep dose–response after 24 h of treatment. To check whether this would also translate to cell proliferation or survival, we treated RMS559 and RH30 cells with NVP-AUY922 and evaluated cell viability after 72 h using the MTS assay. In parallel, we tested the effect of LY2874455 (FGFR inhibitor) and the two inhibitors in combination. HSP90 inhibition efficiently blocked the growth of both cell lines and could therefore be an alternative approach to target FGFRs in rhabdomyosarcoma (Fig. 6d). Interestingly, NVP-AUY922 treatment gave a much steeper dose–response curve than FGFR inhibition in both cell lines. Combination treatment showed stronger activity in RMS559 cells at concentrations below IC_50_ compared to NVP-AUY922 alone. We also evaluated the effect of increasing concentrations of NVP-AUY922 in the presence of a fixed concentration of LY2874455. Since the FGFR inhibitor displayed a different efficacy for the two analysed cell lines, we used 10 nM of LY2874455 for RMS559 cells and 40 nM for RH30 cells. These results also suggested an additive effect of the two drugs (Fig. 6d).

### Susceptibility of FGFR4 V550L signalling to specific FGFR4 inhibitors

Recently, several FGFR4-specific inhibitors have been developed which take advantage of a unique cysteine residue in position 552 close to the active ATP-binding site of the FGFR4 tyrosine kinase [18, 20, 39]. This cysteine is not present in the other FGFRs and therefore offers exceptional specificity to FGFR4.

**Fig. 7:**
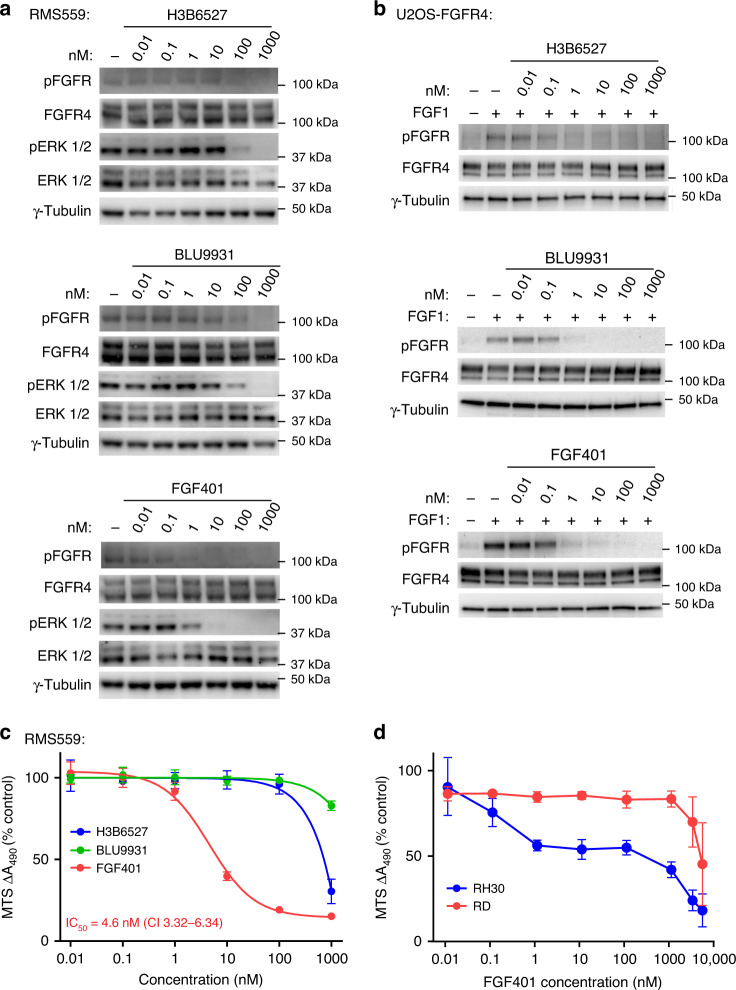
Efficacy of specific FGFR4 inhibitors. **a** RMS559 cells were kept in serum-free media for 1 h prior to treatment with H3B6527, BLU9931 or FGF401 at the indicated concentrations or DMSO (−) for 1 h. Cells were then lysed and the lysates were analysed by western blotting using the indicated antibodies. One representative of three independent experiments for each inhibitor is shown. **b** U2OS-FGFR4 cells were kept in serum-free media for 1 h prior to treatment with H3B6527, BLU9931 or FGF401 at the indicated concentrations or DMSO (−) for 1 h. Thirty minutes before cells were lysed, they were treated with FGF1 (100 ng/ml) in the presence of heparin (20U). The lysates were analysed by western blotting using the indicated antibodies. One representative of three independent experiments for each inhibitor is shown. **c** Efficiency of FGFR4 inhibitors on RMS559 cell viability. RMS559 cells were treated with increasing concentrations of H3B6527, BLU9931 or FGF401 for 6 days before measurement of cell viability using an MTS assay. Data were normalised to the DMSO control and presented as means ± SEM of three independent experiments for H3B6527 and FGF401 and two independent experiments for BLU9931. Curves were fitted with non-linear regression (Hill equation with variable slope), IC_50_ ± 95% CI (confidence interval levels). **d** Efficiency of FGF401 inhibitor on RD cell and RH30 cell viability. RD cells and RH30 cells were treated with increasing concentrations of FGF401 for 6 days before measurement of cell viability using MTS assay. Data were normalised to the DMSO control and presented as means ± SEM of three independent experiments. Outliers were removed according to the 1.5*IQR outlier rule.

To test specific FGFR4 inhibitors against FGFR4 V550L-induced signalling in RMS559 cells, both western blotting and MTS viability assays were performed. By western blotting, we observed that two of the specific FGFR4 inhibitors tested, BLU9931 and H3B6527, inhibited FGFR4 V550L signalling poorly (Fig. 7a). The V550L gatekeeper mutation probably hinders the binding of the drugs to the active site. In accordance with this, both inhibitors displayed very potent inhibition of wild-type FGFR4 tested in U2OS cells stably expressing wild-type FGFR4 (Fig. 7b). Interestingly, FGF401, another FGFR4-specific inhibitor, was very potent and exhibited good inhibition of FGFR4 signalling at low nM concentrations in both RMS559 cells and U2OS-FGFR4 cells (Fig. 7a, b).

These results were reflected in MTS viability assays showing that BLU9931 and H3B6527 had little effect on RMS559 cell viability at nM concentrations, while FGF401 treatment reduced viability already at low nM concentrations with an IC_50_ of 4.6 nM (Fig. 7c). We also tested FGF401 in two other RMS cell lines harbouring wild-type FGFR4 (RH30 and RD). While there was very little effect in RD cells, FGF401 showed some activity on RH30 cell viability (Fig. 7d). However, RMS559 was clearly more sensitive to FGF401 than the two other cell lines. Thus, we conclude that FGF401 could potentially be an efficient inhibitor of mutant FGFR4 V550L signalling in RMS patients.

### FGF401 inhibits the growth of RMS559 xenografts

To assess the in vivo efficacy and clinical utility of FGFR4 V550L targeting, we injected RMS559 cells subcutaneously in the flanks of athymic nude mice. The cells readily formed tumours, and we proceeded to test the in vivo efficacy of LY2874455 and FGF401 in mice bearing RMS559 xenografts.

**Fig. 8:**
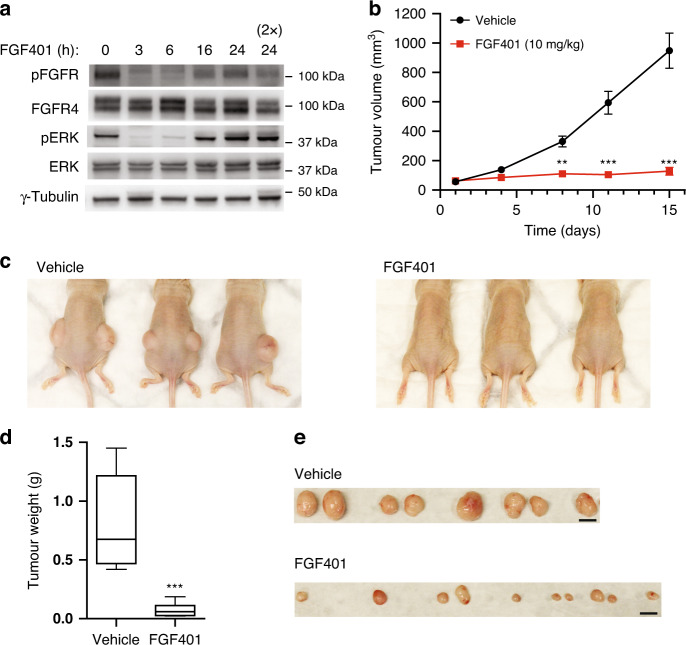
FGF401 blocks RMS559 cell growth in vivo. **a** RMS559 cells were injected on the flanks of female athymic nude mice. When tumours had grown to 300–400 mm^3^ in size, the mice were treated orally with 10 mg/kg FGF401 or vehicle and sacrificed at 3, 6, 16 and 24 h after treatment. In one case, FGF401 was administered twice within the 24 h period (indicated with 2×). Protein lysates were isolated from the tumours and subjected to western blot analysis. A p in front of the name of the antibody indicates that it recognises the phosphorylated form of the protein. **b** Tumour volume of mice as a function of time. RMS559 cells were injected on the flanks of female athymic nude mice. When tumours had grown to 60 mm^3^ in size, the mice were treated orally with 10 mg/kg FGF401 or vehicle. Tumour diameter was measured twice per week and the tumour volume was calculated (0.5 × length × width^2^). The experiment was terminated when the average tumour volume of the vehicle receiving mice reached 1000 mm^3^. Data are presented as average tumour volume (mm^3^) ± SEM, *n* = 10 for all except *n* = 8 for last time point for vehicle (as one mouse bearing two tumours was sacrificed prior to the rest due to large tumour size). The data were analysed using two-way ANOVA followed by Bonferroni’s post hoc test. ***P < 0.001, **P < 0.01. **c** Images of three representative mice at the end of the experiment for each of the two treatment conditions as described in **b**. **d** Tumour weight of FGF401 or vehicle-treated mice at the end of the experiment as described in **b**. *n* = 10 for FGF401 and *n* = 8 for vehicle. Whiskers, min to max. The data were analysed using an unpaired, two-tailed *t* test. ***P < 0.001. **e** Photos of the tumours excised from the mice treated with FGF401 or vehicle as described in **b**. Scale bar, 1 cm.

First, we analysed by western blotting the ability of the drugs to inhibit FGFR4 signalling after administration to mice. The drug concentrations used were in accordance with previous in vivo studies [19, 32]. After 3–6 h, both drugs efficiently attenuated signalling from FGFR4 V550L in vivo (Fig. 8a and Supplementary Fig. 7a). However, at later time points (16–24 h for FGF401 and 6 h for LY2874455), the FGFR4 signalling activity was partially recovered. For tumour growth measurements utilising RMS559 xenografts, we therefore decided to treat the mice twice daily, except at weekends, where the mice were treated once per day.

To assess the effect of the inhibitors in vivo, the mice were divided into two treatment groups (ten tumours in each). The control group was administered vehicle only, while the other group was either treated by oral gavage with 6 mg/kg LY2874455 or, in another experiment, 10 mg/kg FGF401. LY2874455 showed some activity, but inhibited tumour growth poorly (Supplementary Fig. 7b). In contrast, FGF401 completely inhibited tumour growth in vivo (Fig. 8b–e). The mice suffered negligible weight loss over the treatment time, indicating that both drugs were well-tolerated (Supplementary Fig. 7b–d).

Taken together, the data indicate that FGF410 is orally bioavailable and well-tolerated in RMS559 xenografts and shows promising in vivo activity demonstrating possible future clinical utility.

## Discussion

We have here demonstrated several strategies to inhibit oncogenic signalling from the mutant FGFR4 V550L: (1) The pan-FGFR inhibitor LY2874455 reduces signalling activity of FGFR4 V550L and inhibits RMS viability; (2) Inhibitors targeting HSP90 (NVP-AUY922) destabilise and inhibit FGFR4 mutants; (3) Inhibitors of FGFR4 downstream signalling pathways RAS/MAPK and PI3K/AKT (e.g.,TAK733 and PI-103) antagonise FGFR4-induced proliferation; (4) A gatekeeper agnostic FGFR4 inhibitor (FGF401) has potent activity against FGFR4 V550L signalling and efficiently inhibits tumour growth in vivo in mice injected with RMS559 cells.

The V550L mutation is located close to the ATP-binding site of the kinase, which is also the binding site for currently available FGFR kinase inhibitors. Mutation of the Val 550 residue is a gatekeeper mutation conferring resistance to several of the FGFR inhibitors developed so far (e.g., erdafitinib). However, it was recently shown that LY2874455 can inhibit FGFR4 with gatekeeper mutation [38]. We also show that LY2874455 inhibits FGFR4 V550L in RMS cells, although LY2874455 was less active than FGF401, particularly in vivo.

Recently, several inhibitors targeting FGFR4 have been developed. These inhibitors act by reversibly or irreversibly binding to Cys 552 in the catalytic domain of the FGFR4 kinase. It is therefore not surprising that Val 550 mutations hamper the activity of some of these inhibitors. In the case of Filgotinib (BLU554), which is closely related to BLU9931, FGFR4 V550L mutations caused resistance to the drug in a clinical trial for hepatic cancer [38], clearly implicating FGFR4 as a driver of this cancer and V550L mutations as gate-keepers for the drug. Roblitinib (FGF401) is also in clinical trials against hepatic cancer and has shown promising anti-tumour activity [18, 19]. FGF401 has previously been shown to have reduced activity against the FGFR4 V550E mutant [19]. However, Zhou et al. demonstrated that FGF401 has activity in vitro against FGFR4 V550L comparable to wild-type FGFR4 [40]. We found here that FGF401 has high activity against FGFR4 V550L. Possibly the larger size of Glu compared to Leu could explain the discrepancy between these studies as a more bulky amino acid like Glu would more efficiently block the binding site. This also implies that it is important to consider which amino acid Val 550 is mutated to, when choosing an appropriate inhibitor. Clearly, FGFR4 V550L can be targeted by FGF401, but more studies are needed to clarify if the FGFR4 V550E/M mutations are also sensitive to this drug. H3B6527 has not previously been tested against mutant FGFR4 [39], but our results indicate that the inhibitor is blocked by mutations in Val 550, resulting in reduced activity.

Although PTPN11 inhibitors have shown promising effects in some cancers driven by aberrant RTK signalling or activated PTPN11, we did not observe a reduction of RMS559 viability when treating the cells with the SHP099 inhibitor. Possibly, the MAPK pathway is activated also independently of PTPN11 in RMS559 cells. Interestingly, it was previously shown that SHP099 had little effect in cancer cells driven by FGFR signalling, because of compensatory feedback activation of FGFR [41].

HSP90 inhibitors had a strong effect on the stability of FGFR4 V550L causing efficient down-regulation of signalling and cell viability. However, this effect was not specific to mutant FGFR4 as we also observed degradation of wild-type FGFR4 and decreased cell viability in RH30 cells. As wild-type FGFR4 has been implicated in fusion-positive RMS cell survival [13], these findings provide a rationale for HSP90 inhibitors in fusion-positive RMSs, as well. Clearly, HSP90 has many important client proteins that could be involved in the proliferation and survival of cancer cells. Many kinases are dependent on HSP90 through the co-chaperone Cdc37, including MAPK and PI3K pathway kinases [42]. Therefore targeting HSP90 will potentially exert additive or synergistic effects on FGFR4-dependent RMS, affecting both upstream and downstream signalling components. It is likely that additional targets in addition to FGFR4 are responsible for the strong inhibition observed in RMS559 cells.

Interestingly, we found that both the RAS/MAPK and PI3K/AKT pathways contribute to RMS559 cell viability. RMS559 cells seem to be highly sensitive to PI3K inhibitors as the IC_50_ of PI-103 (see Fig. 5) are in the range of previously reported highly sensitive cells [43]. The effect of MAPK inhibitors seems more modest, as their IC_50_s (Fig. 5) are higher than what is observed in highly sensitive cells [44–46]. This is in agreement with McKinnon et al., who found that inhibitors of both pathways reduced viability in a screen using cells derived from transgenic mice expressing mutant FGFR4, but inhibitors of PI3K/mTOR were particularly effective [14].

In addition to RMS and the abovementioned clinical evidence in hepatocellular carcinoma, the FGFR4 V550L mutation has been reported in several other cancer types. For example, 3% of desmoplastic small round cell tumours have FGFR4 V550L mutations [47], and 3.5% of endocrine-treated metastatic invasive lobular breast carcinomas harbour FGFR4 mutations, including N535K/D and V550L/M [48]. Our RMS studies provide further rationale for treating subsets of cancer with inhibitors tailored to particular FGFR4 oncogenic mutations.

A potential limitation of these studies is the focus on cell cultures and xenografts from one RMS model driven by V550L mutation. As we enter the new era of precision medicine in the clinic, research based on several relevant models, when feasible, is important due to the intrinsic biologic heterogeneity in many cancers. However, RMS is a rare disease, and RMS559 is the only patient-derived cell line with FGFR4 V550L oncogenic mutation. Fortunately, paediatric RMS have few mutations per case [7], such that molecular and biologic heterogeneity is less likely to confound interpretations based on one model than in genomically complex sarcomas such as osteosarcoma or leiomyosarcoma. We also note that dramatic therapeutic progress in other sarcomas, particularly in GIST (which are also genomically noncomplex), was achieved with the use of a single, representative, model of oncogenic KIT-dependency [49].

While rhabdomyosarcoma (RMS) has a high probability of positive treatment outcome, many patients with metastatic disease succumb to their disease and patients with relapse usually have a dismal prognosis. Furthermore, the high response rates in childhood cancer come at a cost, as many paediatric cancer patients suffer from severe side effects later in life. It is therefore clear that new treatment strategies are required to improve the outcome of paediatric cancer patients. We have here presented several promising strategies that warrant further preclinical and clinical testing giving new possibilities to treat RMS driven by FGFR4 alterations.

## Supplementary information

Checklist

Supplementary Figure Legends

Table S1

Supplementary Figures

## Data Availability

The mass spectrometry proteomics data have been deposited to the ProteomeXchange Consortium via the PRIDE [50] partner repository with the dataset identifier PXD029719. All other datasets are available upon request.

## References

[1] Ognjanovic S, Linabery AM, Charbonneau B, Ross JA (2009). Trends in childhood rhabdomyosarcoma incidence and survival in the United States, 1975-2005. Cancer.

[2] Skapek SX, Ferrari A, Gupta AA, Lupo PJ, Butler E, Shipley J (2019). Rhabdomyosarcoma. Nat Rev Dis Prim.

[3] Huh WW, Skapek SX (2010). Childhood rhabdomyosarcoma: new insight on biology and treatment. Curr Oncol Rep..

[4] Tonin PN, Scrable H, Shimada H, Cavenee WK (1991). Muscle-specific gene expression in rhabdomyosarcomas and stages of human fetal skeletal muscle development. Cancer Res.

[5] Soleimani VD, Rudnicki MA (2011). New insights into the origin and the genetic basis of rhabdomyosarcomas. Cancer Cell.

[6] El Demellawy D, McGowan-Jordan J, de Nanassy J, Chernetsova E, Nasr A (2017). Update on molecular findings in rhabdomyosarcoma. Pathology.

[7] Shern JF, Chen L, Chmielecki J, Wei JS, Patidar R, Rosenberg M (2014). Comprehensive genomic analysis of rhabdomyosarcoma reveals a landscape of alterations affecting a common genetic axis in fusion-positive and fusion-negative tumors. Cancer Discov.

[8] Taylor JG, Cheuk AT, Tsang PS, Chung JY, Song YK, Desai K (2009). Identification of FGFR4-activating mutations in human rhabdomyosarcomas that promote metastasis in xenotransplanted models. J Clin Investig.

[9] Wesche J, Haglund K, Haugsten EM (2011). Fibroblast growth factors and their receptors in cancer. Biochemical J.

[10] Marics I, Padilla F, Guillemot JF, Scaal M, Marcelle C (2002). FGFR4 signaling is a necessary step in limb muscle differentiation. Development.

[11] Marcelle C, Wolf J, Bronner-Fraser M (1995). The in vivo expression of the FGF receptor FREK mRNA in avian myoblasts suggests a role in muscle growth and differentiation. Developmental Biol.

[12] Zhao P, Caretti G, Mitchell S, McKeehan WL, Boskey AL, Pachman LM (2006). Fgfr4 is required for effective muscle regeneration in vivo. Delineation of a MyoD-Tead2-Fgfr4 transcriptional pathway. J Biol Chem.

[13] Crose LE, Etheridge KT, Chen C, Belyea B, Talbot LJ, Bentley RC, et al. FGFR4 blockade exerts distinct antitumorigenic effects in human embryonal versus alveolar rhabdomyosarcoma. Clin Cancer Res. 2012;18:3780–90. 10.1158/1078-0432.CCR-10-3063 PMC3713717 22648271

[14] McKinnon T, Venier R, Yohe M, Sindiri S, Gryder BE, Shern JF (2018). Functional screening of FGFR4-driven tumorigenesis identifies PI3K/mTOR inhibition as a therapeutic strategy in rhabdomyosarcoma. Oncogene.

[15] Li SQ, Cheuk AT, Shern JF, Song YK, Hurd L, Liao H (2013). Targeting wild-type and mutationally activated FGFR4 in rhabdomyosarcoma with the inhibitor ponatinib (AP24534). PLoS ONE.

[16] Beenken A, Mohammadi M (2009). The FGF family: biology, pathophysiology and therapy. Nat Rev Drug Discov.

[17] Babina IS, Turner NC (2017). Advances and challenges in targeting FGFR signalling in cancer. Nat Rev Cancer.

[18] Fairhurst RA, Knoepfel T, Buschmann N, Leblanc C, Mah R, Todorov M (2020). Discovery of roblitinib (FGF401) as a reversible-covalent inhibitor of the kinase activity of fibroblast growth factor receptor 4. J Medicinal Chem.

[19] Weiss A, Adler F, Buhles A, Stamm C, Fairhurst RA, Kiffe M (2019). FGF401, a first-in-class highly selective and potent FGFR4 inhibitor for the treatment of FGF19-driven hepatocellular cancer. Mol Cancer Ther.

[20] Hagel M, Miduturu C, Sheets M, Rubin N, Weng W, Stransky N (2015). First selective small molecule inhibitor of FGFR4 for the treatment of hepatocellular carcinomas with an activated FGFR4 signaling pathway. Cancer Discov.

[21] Wesche J, Malecki J, Wiedlocha A, Ehsani M, Marcinkowska E, Nilsen T (2005). Two nuclear localization signals required for transport from the cytosol to the nucleus of externally added FGF-1 translocated into cells. Biochemistry.

[22] Haugsten EM, Malecki J, Bjorklund SM, Olsnes S, Wesche J (2008). Ubiquitination of fibroblast growth factor receptor 1 is required for its intracellular sorting but not for its endocytosis. Mol Biol Cell.

[23] McLaren W, Gil L, Hunt SE, Riat HS, Ritchie GR, Thormann A (2016). The ensembl variant effect predictor. Genome Biol.

[24] Lever J, Zhao EY, Grewal J, Jones MR, Jones SJM (2019). CancerMine: a literature-mined resource for drivers, oncogenes and tumor suppressors in cancer. Nat Methods.

[25] Repana D, Nulsen J, Dressler L, Bortolomeazzi M, Venkata SK, Tourna A (2019). The Network of Cancer Genes (NCG): a comprehensive catalogue of known and candidate cancer genes from cancer sequencing screens. Genome Biol.

[26] Sondka Z, Bamford S, Cole CG, Ward SA, Dunham I, Forbes SA (2018). The COSMIC Cancer Gene Census: describing genetic dysfunction across all human cancers. Nat Rev Cancer.

[27] Nakken S, Fournous G, Vodak D, Aasheim LB, Myklebost O, Hovig E (2018). Personal cancer genome reporter: variant interpretation report for precision oncology. Bioinformatics.

[28] Karczewski KJ, Francioli LC, Tiao G, Cummings BB, Alföldi J, Wang Q (2020). The mutational constraint spectrum quantified from variation in 141,456 humans. Nature.

[29] Sukhai MA, Misyura M, Thomas M, Garg S, Zhang T, Stickle N (2019). Somatic tumor variant filtration strategies to optimize tumor-only molecular profiling using targeted next-generation sequencing panels. J Mol Diagnos: JMD.

[30] Batth TS, Tollenaere MX, Rüther P, Gonzalez-Franquesa A, Prabhakar BS, Bekker-Jensen S (2019). Protein aggregation capture on microparticles enables multipurpose proteomics sample preparation. Mol Cell Proteom.

[31] Cox J, Mann M (2008). MaxQuant enables high peptide identification rates, individualized p.p.b.-range mass accuracies and proteome-wide protein quantification. Nat Biotechnol.

[32] Zhao G, Li WY, Chen D, Henry JR, Li HY, Chen Z (2011). A novel, selective inhibitor of fibroblast growth factor receptors that shows a potent broad spectrum of antitumor activity in several tumor xenograft models. Mol Cancer Ther.

[33] Shukla N, Ameur N, Yilmaz I, Nafa K, Lau CY, Marchetti A (2012). Oncogene mutation profiling of pediatric solid tumors reveals significant subsets of embryonal rhabdomyosarcoma and neuroblastoma with mutated genes in growth signaling pathways. Clin Cancer Res.

[34] Sun X, Ren Y, Gunawan S, Teng P, Chen Z, Lawrence HR (2018). Selective inhibition of leukemia-associated SHP2(E69K) mutant by the allosteric SHP2 inhibitor SHP099. Leukemia.

[35] Tate JG, Bamford S, Jubb HC, Sondka Z, Beare DM, Bindal N (2019). COSMIC: the catalogue of somatic mutations in cancer. Nucleic Acids Res.

[36] Uhlén M, Fagerberg L, Hallström BM, Lindskog C, Oksvold P, Mardinoglu A (2015). Tissue-based map of the human proteome. Science.

[37] Huang Z, Tan L, Wang H, Liu Y, Blais S, Deng J (2015). DFG-out mode of inhibition by an irreversible type-1 inhibitor capable of overcoming gate-keeper mutations in FGF receptors. ACS Chem Biol.

[38] Hatlen MA, Schmidt-Kittler O, Sherwin CA, Rozsahegyi E, Rubin N, Sheets MP (2019). Acquired on-target clinical resistance validates FGFR4 as a driver of hepatocellular carcinoma. Cancer Discov.

[39] Joshi JJ, Coffey H, Corcoran E, Tsai J, Huang CL, Ichikawa K (2017). H3B-6527 is a potent and selective inhibitor of FGFR4 in FGF19-driven hepatocellular carcinoma. Cancer Res.

[40] Zhou Z, Chen X, Fu Y, Zhang Y, Dai S, Li J (2019). Characterization of FGF401 as a reversible covalent inhibitor of fibroblast growth factor receptor 4. Chem Commun.

[41] Lu H, Liu C, Huynh H, Le TBU, LaMarche MJ, Mohseni M (2020). Resistance to allosteric SHP2 inhibition in FGFR-driven cancers through rapid feedback activation of FGFR. Oncotarget.

[42] Calderwood SK (2015). Cdc37 as a co-chaperone to Hsp90. Sub-Cell Biochem.

[43] Raynaud FI, Eccles S, Clarke PA, Hayes A, Nutley B, Alix S (2007). Pharmacologic characterization of a potent inhibitor of class I phosphatidylinositide 3-kinases. Cancer Res.

[44] Dong Q, Dougan DR, Gong X, Halkowycz P, Jin B, Kanouni T (2011). Discovery of TAK-733, a potent and selective MEK allosteric site inhibitor for the treatment of cancer. Bioorg medicinal Chem Lett.

[45] von Euw E, Atefi M, Attar N, Chu C, Zachariah S, Burgess BL (2012). Antitumor effects of the investigational selective MEK inhibitor TAK733 against cutaneous and uveal melanoma cell lines. Mol Cancer.

[46] Ciuffreda L, Del Bufalo D, Desideri M, Di Sanza C, Stoppacciaro A, Ricciardi MR (2009). Growth-inhibitory and antiangiogenic activity of the MEK inhibitor PD0325901 in malignant melanoma with or without BRAF mutations. Neoplasia.

[47] Slotkin EK, Bowman AS, Levine MF, Dela Cruz F, Coutinho DF, Sanchez GI (2021). Comprehensive molecular profiling of desmoplastic small round cell tumor. Mol Cancer Res: MCR.

[48] Levine KM, Priedigkeit N, Basudan A, Tasdemir N, Sikora MJ, Sokol ES (2019). FGFR4 overexpression and hotspot mutations in metastatic ER+ breast cancer are enriched in the lobular subtype. npj Breast Cancer.

[49] Tuveson DA, Willis NA, Jacks T, Griffin JD, Singer S, Fletcher CD (2001). STI571 inactivation of the gastrointestinal stromal tumor c-KIT oncoprotein: biological and clinical implications. Oncogene.

[50] Perez-Riverol Y, Csordas A, Bai J, Bernal-Llinares M, Hewapathirana S, Kundu DJ (2019). The PRIDE database and related tools and resources in 2019: improving support for quantification data. Nucleic Acids Res.

