# Supplementary material for: Strategies to inhibit FGFR4 V550L-driven rhabdomyosarcoma
Source: Br J Cancer. 2022 Sep 12;127(11):1939–53. doi: 10.1038/s41416-022-01973-6 (PMC9681859; doi:10.1038/s41416-022-01973-6)
Supplement: Supplementary file 2 — Supplementary Figure Legends [file 41416_2022_1973_MOESM2_ESM.docx]

## Supplementary Figure Legends

**Supplementary Figure 1.** Expression levels of FGFR4 downstream adaptors and effectors in RMS559 cells.

**Supplementary Figure 2.** Low magnification images of different cells from the same experiment as the images presented in Figure 3. In brief, the RMS559 cells growing on coverslips were transfected with non-coding, control siRNAs (scr) or siRNAs targeting FGFR4 (siR4 #2) for 48 hours. Then the cells were left untreated (-) or incubated with DL550-FGF1 (FGF1) and heparin (50 U/ml) for 30 minutes. The cells were then fixed and stained with anti-FGFR4 (**a**) or anti-pFGFR (**b**) antibodies and Hoechst and analysed by confocal microscopy. Images were taken at fixed intensity settings, and brightness/contrast was adjusted equally for all images. Scale bar, 20 µm.

**Supplementary Figure 3.** (**a**) Western blot demonstrating the efficiency of LY2874455 (100 nM) and FGF1 (100 ng/ml) treatment of the samples subjected to phosphoproteomics. (**b**) Enriched canonical pathways were investigated by the Ingenuity Pathway Analysis (IPA) software using the dataset predicted to be dependent on FGFR signalling.

**Supplementary Figure 4.** Efficacy of RAS/MAPK pathway inhibitors in RMS559 cells. The efficiency of the MAPK pathway inhibitors TAK632 (**a**), RO51267663 (**b**) or U0126 (**c**) in RMS559 cells were examined by cell viability MTS assays (left) and western blots (right). For MTS assays, RMS559 cells were treated with increasing concentrations of the inhibitors (as indicated) for six days before measurement of cell viability using the MTS assay. Data was normalized to the DMSO control and presented as mean ± SEM of at least three independent experiments (except RO51267663 0.03 µM and U0126 0.3 µM, which were performed twice). Note the different scales on the x-axes (log2). For western blotting, RMS559 cells were kept in serum-free media for one hour prior to one hour incubation with DMSO (-) or increasing concentrations of the inhibitors. Cells were then lysed and analysed by Western blotting using the indicated antibodies. One representative experiment of at least two for each inhibitor is shown.

**Supplementary Figure 5.** Efficacy of PI3K/AKT pathway and PTPN11 inhibitor in RMS559 cells. The efficiency of the PI3K pathway inhibitor, LY294002 **(a)** or PTPN11 inhibitor, SHP099 **(b)** in RMS559 were checked in MTS assays (left) and western blots (right). U2OS-FGFR4 was also used to test the efficiency of SHP099 **(c)**. For MTS assays, RMS559 cells were treated with increasing concentrations of the inhibitors (as indicated) for six days before measurement of cell viability using the MTS assay. Data was normalized to the DMSO control and presented as means ± SEM of three independent experiments for LY294002 and two for SHP099. Note the different scales on the x-axes (log2). For western blotting, the cells were kept in serum-free media for one hour prior to one hour incubation with DMSO (-) or increasing concentrations of the inhibitors. Cells were then lysed and analysed by Western blotting using the indicated antibodies. One representative experiment of at least two for each inhibitor is shown.

**Supplementary Figure 6.** RMS559 and RH30 cells were treated with increasing concentrations of NVP-AUY922, an HSP90 inhibitor, for 24 hours and then lysed. The effect of inhibitor treatment was assessed in western blotting using denoted antibodies. One representative experiment of two is shown.

**Supplementary Figure 7.** Efficiency of LY2874455 treatment on RMS559 cell growth *in vivo***. (a)** RMS559 cells were injected on the flanks of female athymic nude mice. When tumours had grown to 300-400 mm^3^ in size the mice were treated orally with 1.5-6 mg/kg LY2874455 or vehicle and sacrificed 3 or 6 hours after treatment. Protein lysates were isolated from the tumours and subjected to western blot analysis. **(b)** Tumour volume of mice as a function of time. RMS559 cells were injected on the flanks of female athymic nude mice. When tumours had grown to 60 mm^3^ in size the mice were treated orally with 6 mg/kg LY2874455 or vehicle and tumour diameter was measured twice per week and the tumour volume was calculated (0.5 x length x width^2^). The experiment was terminated when the tumour volume of the vehicle receiving mice reached 1000 mm^3^. Data is presented as average tumour volume (mm^3^) ± SEM, n=11 for vehicle and 10 for LY2874455 treatment until day 15. The experiment was ended at this stage for mice bearing the largest tumours. On day 18, n=1 for vehicle and n=7 for LY2874455. **(c)** Bodyweight of mice treated with 6 mg/kg LY2874455 or vehicle as described in **b**. **(d)** Bodyweight of mice treated with 10 mg/kg FGF401 or vehicle as described in Fig. 8b.
