# Supplementary material for: Strategies to inhibit FGFR4 V550L-driven rhabdomyosarcoma
Source: Br J Cancer. 2022 Sep 12;127(11):1939–53. doi: 10.1038/s41416-022-01973-6 (PMC9681859; doi:10.1038/s41416-022-01973-6)
Supplement: Supplementary file 3 — Table S1 [file 41416_2022_1973_MOESM3_ESM.docx]

| **Protein**  **name** | **Protein**  **activity** | **Site** | **Upstream kinase** | **RTK**  **induced*** | **FGFR**  **induced*** | **Fold**  **increase** |
| --- | --- | --- | --- | --- | --- | --- |
| MARCKS | Actin crosslinking | S170 | PKCA | + |  | 180 |
| ERK2 | Kinase | T185 | MEK1 | + | + | 114 |
| RANBP2; RGPD4 | E3 SUMO ligase | S2276;  S1301 | CDK1 | + |  | 99 |
| CHD4 | DNA helicase | T1540 |  |  |  | 86 |
| NCBP1 | RNA regulator | S22 | P70S6K | + |  | 78 |
| CHD2 | DNA helicase | S1213 |  |  |  | 76 |
| Paxillin | Focal adhesion signaling | S244 | CDK5 |  |  | 67 |
| RRM2 | DNA replication | S20 | CDK1/2 |  |  | 60 |
| RANBP2;RGPD4 | E3 SUMO ligase | S2280;  S1305 | CDK1 | + |  | 55 |
| NOL5A | rRNA processing | S519 |  |  |  | 54 |
| TACC2 | Microtubule coupling of nucleus and centrosome | S2321 |  |  |  | 47 |
| Histone 2 H3A;C;D | Nucleosome component | S29;S29;S29 |  |  |  | 46 |
| BAG3 | Co-chaperone | S377 |  | + |  | 37 |
| Nogo | ER formation | S7 |  |  |  | 25 |
| Nestin | Disassembly of phosphorylated vimentin IF | T384 |  |  |  | 23 |
| PHLDB2 | Microtubule organization | T469 |  |  |  | 21 |
| SPT6 | Transcription elongation factor | S1535 |  |  |  | 21 |
| Lamin A | Nuclear lamina component | S403 |  |  |  | 19 |
| N-PAC |  | S130 |  |  |  | 17 |
| FLYWCH2 |  | S21 |  |  |  | 16 |
| TOR1AIP1 | Nuclear membrane integrity | S215 |  |  |  | 16 |
| BCDIN3 | RNA metyltransferase | S152 |  |  |  | 15 |

**Table S1.** Top 20 serine/threonine phosphorylation sites induced by FGFR4 V550L in RMS559 cells. The phosphorylation sites were identified by phosphoproteomics. The information regarding the phosphorylation sites were found on [www.phosphosite.org](http://www.phosphosite.org). IF, intermediate filaments, *Phosphorylation induced or repressed by stimulation or inhibition, respectively of RTK/FGFR pathway.
