# Supplementary material for: Strategies to inhibit FGFR4 V550L-driven rhabdomyosarcoma
Source: Br J Cancer. 2022 Sep 12;127(11):1939–53. doi: 10.1038/s41416-022-01973-6 (PMC9681859; doi:10.1038/s41416-022-01973-6)

Supplementary Figure 1

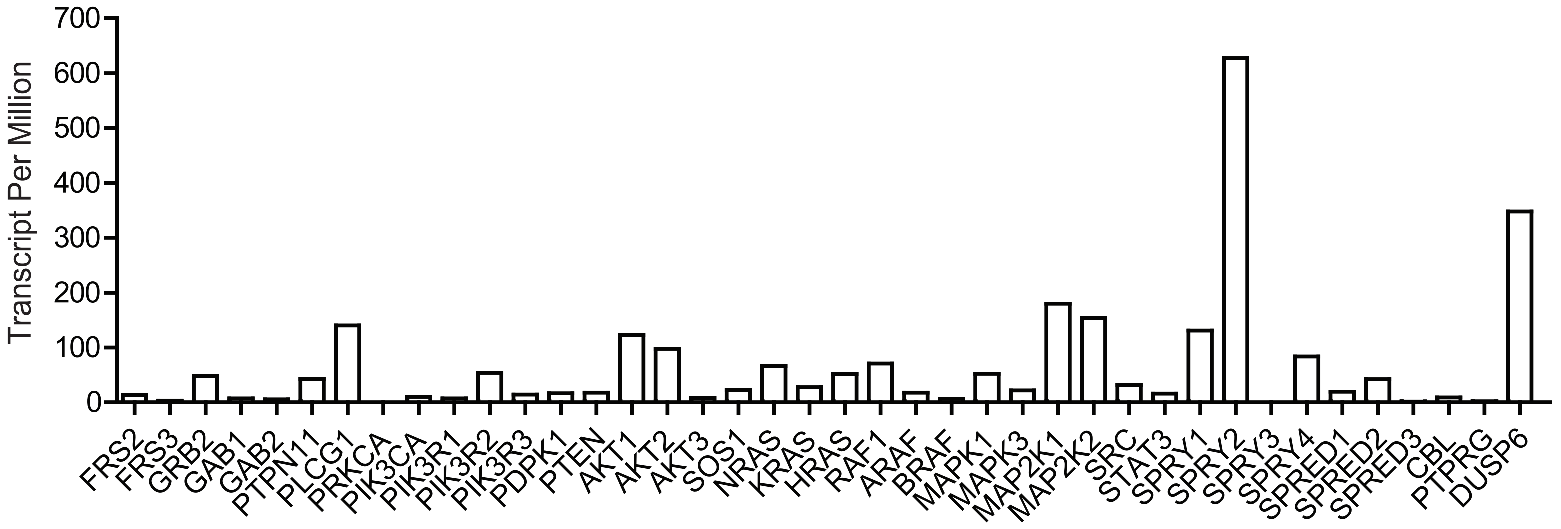

Supplementary Figure 2

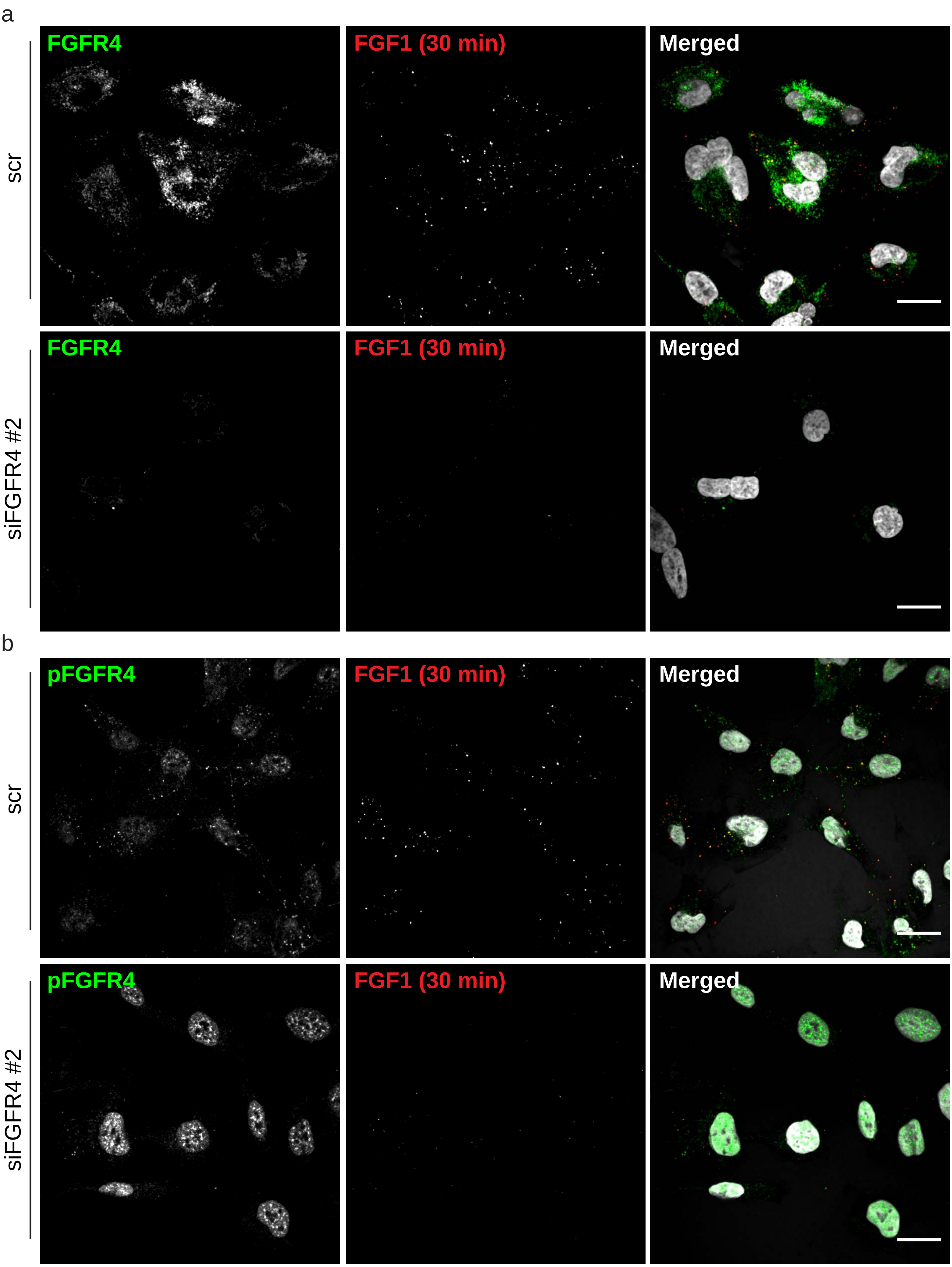

Supplementary Figure 3

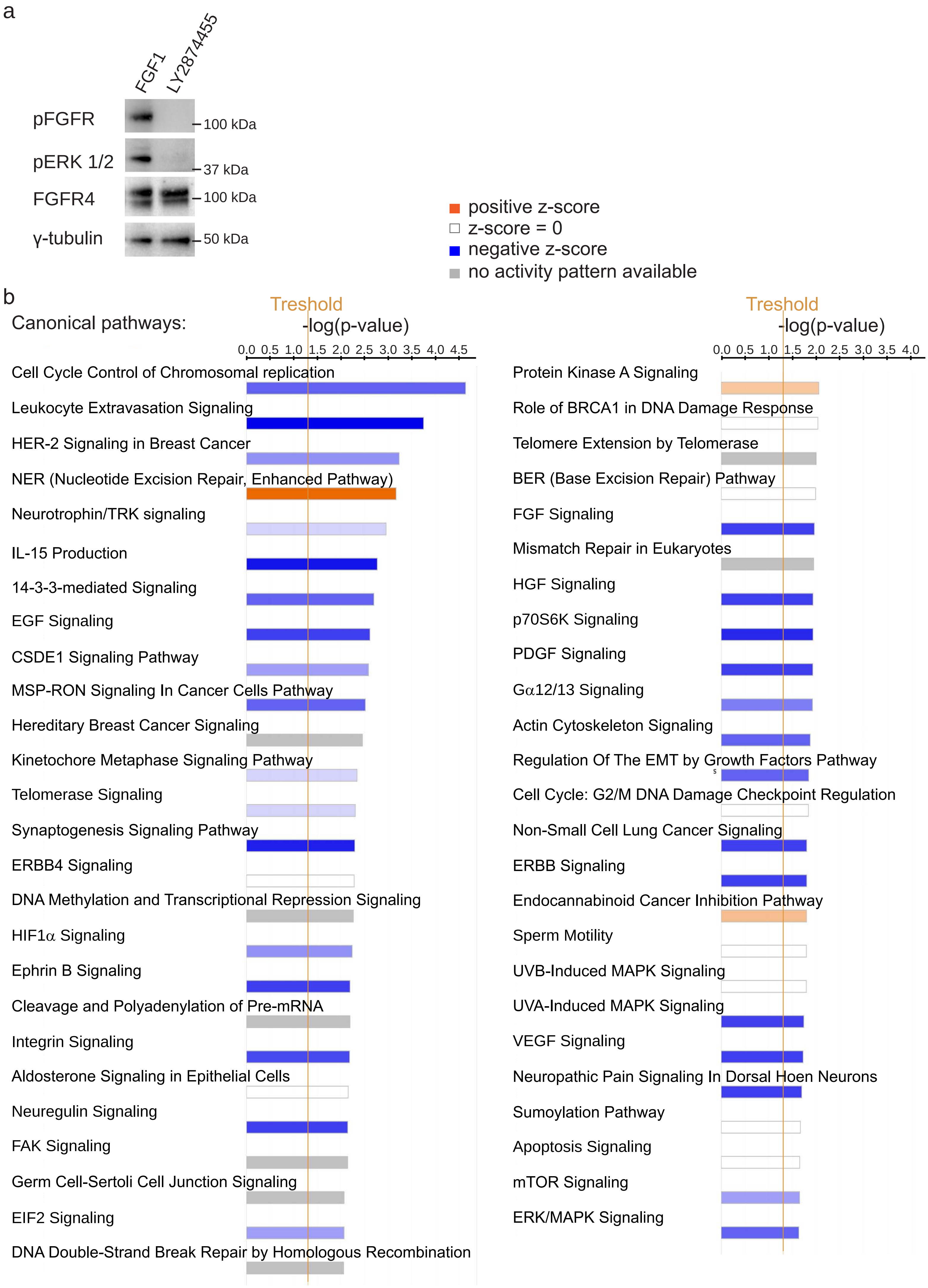

Supplementary Figure 4

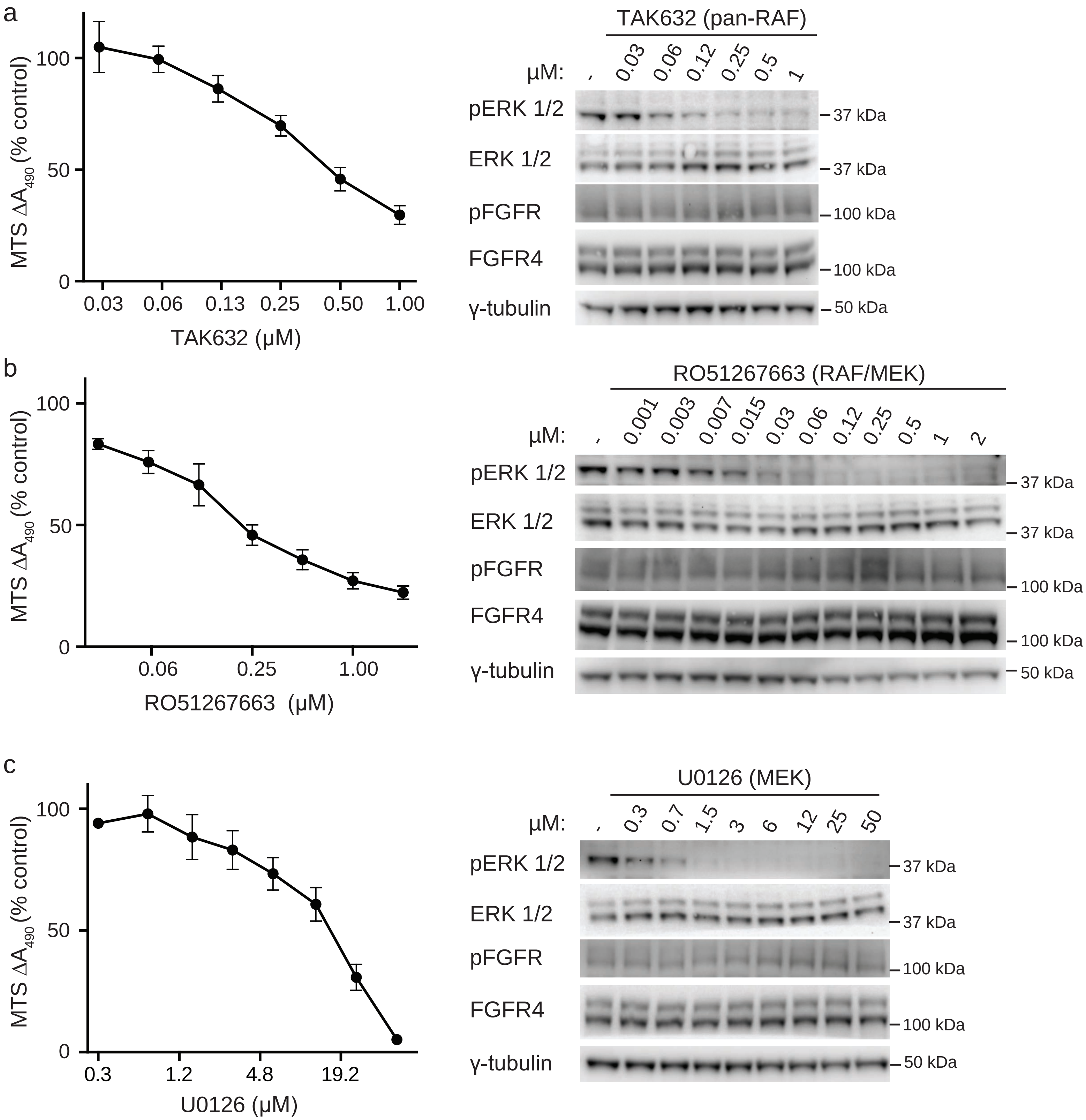

Supplementary Figure 5

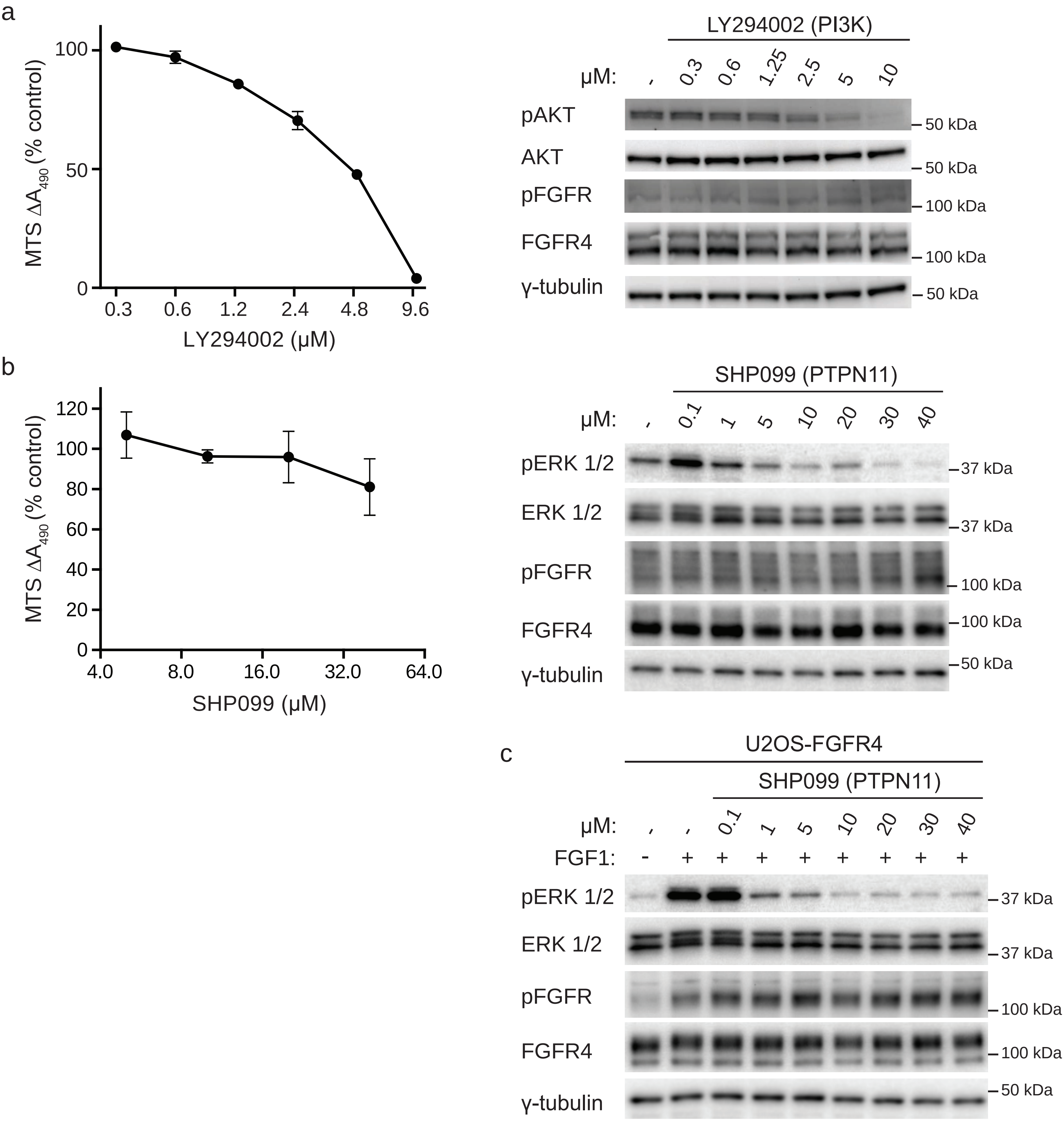

Supplementary Figure 6

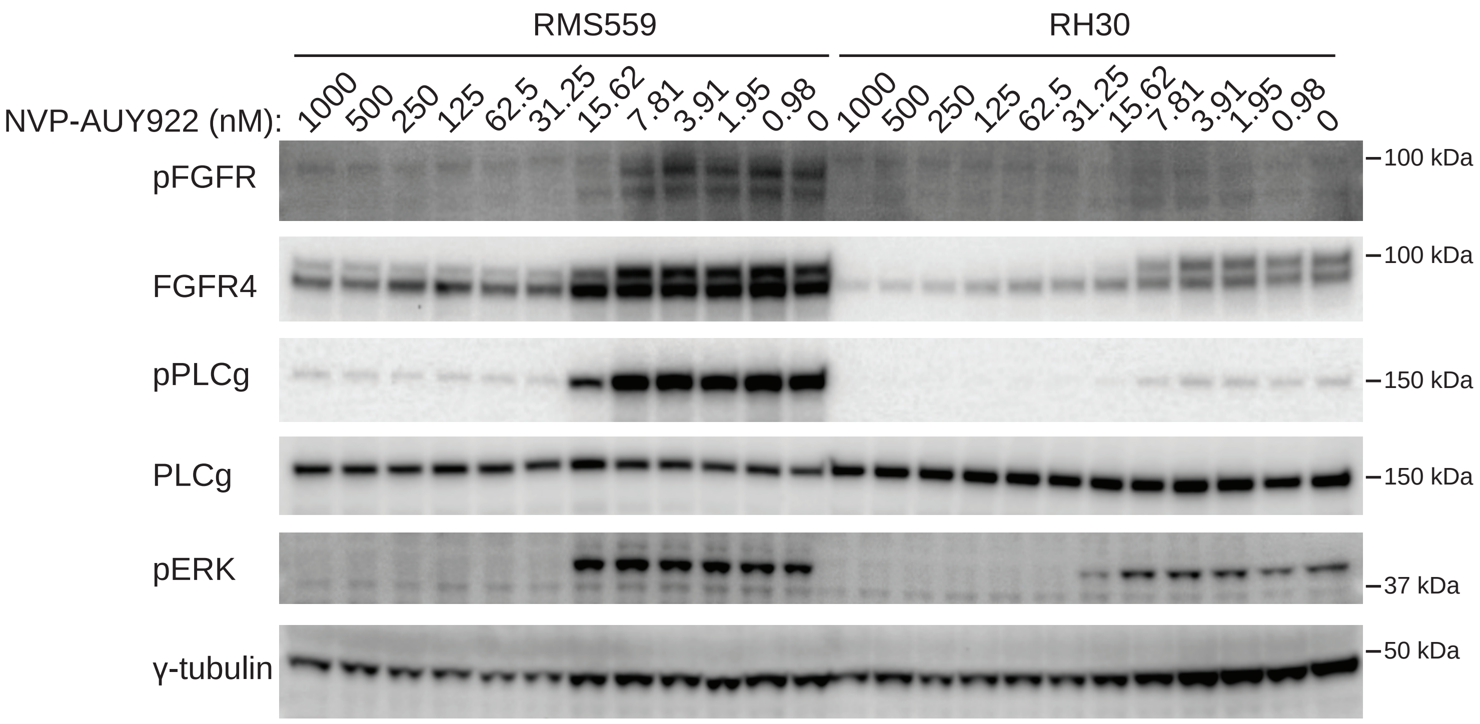

Supplementary Figure 7

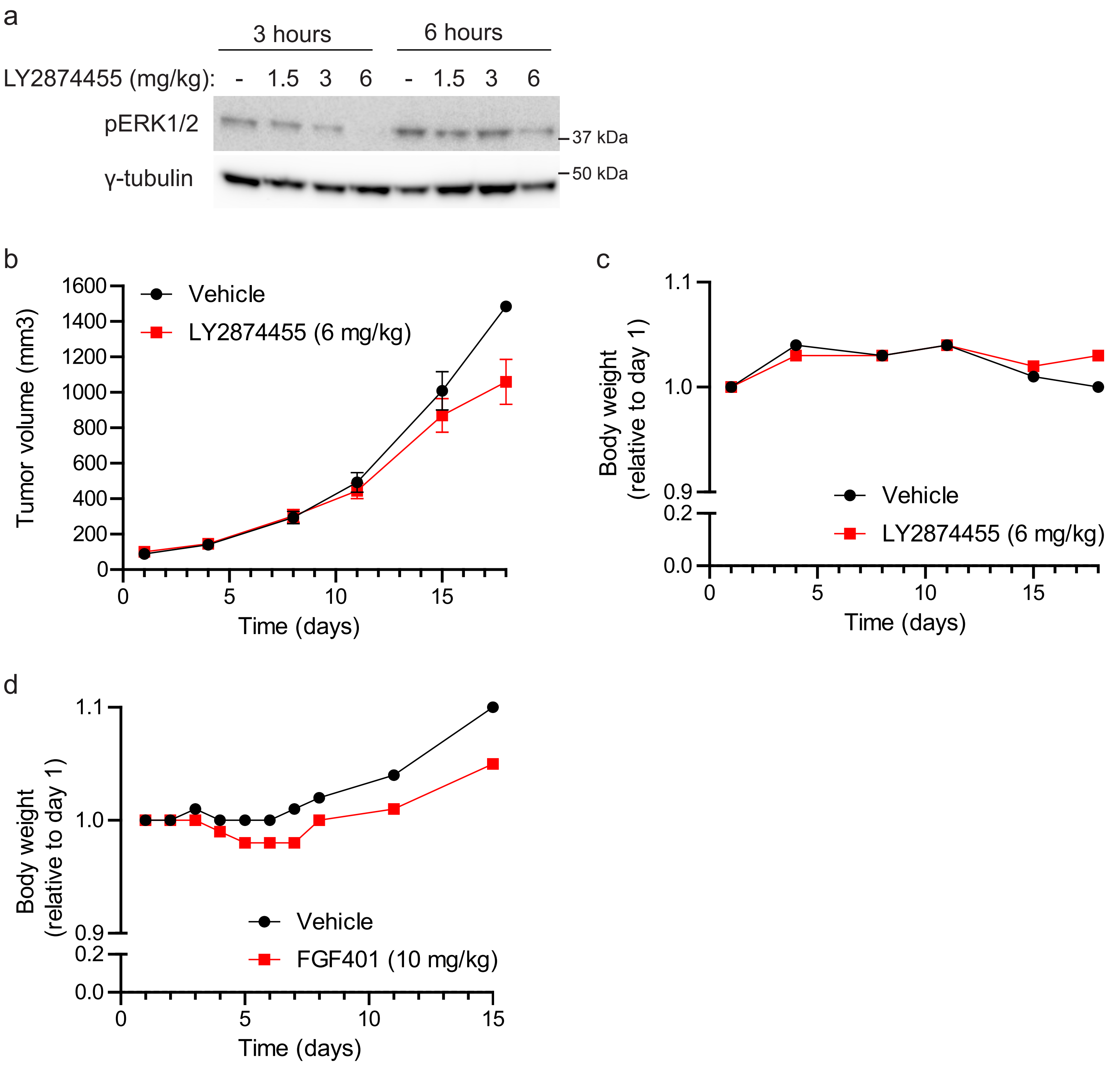

Supplement: Supplementary file 4 — Supplementary Figures [file 41416_2022_1973_MOESM4_ESM.pdf]
